# Pinaceae Pine Resins (Black Pine, Shore Pine, Rosin, and Baltic Amber) as Natural Dielectrics for Low Operating Voltage, Hysteresis‐Free, Organic Field Effect Transistors

**DOI:** 10.1002/gch2.202300062

**Published:** 2023-09-06

**Authors:** Maria Elisabetta Coppola, Andreas Petritz, Cristian Vlad Irimia, Cigdem Yumusak, Felix Mayr, Mateusz Bednorz, Aleksandar Matkovic, Muhammad Awais Aslam, Klara Saller, Clemens Schwarzinger, Maria Daniela Ionita, Manuela Schiek, Annika I. Smeds, Yolanda Salinas, Oliver Brüggemann, Rosarita D'Orsi, Marco Mattonai, Erika Ribechini, Alessandra Operamolla, Christian Teichert, Chunlin Xu, Barbara Stadlober, Niyazi Serdar Sariciftci, Mihai Irimia‐Vladu

**Affiliations:** ^1^ Joanneum Research Forschungsgesellschaft Materials Franz‐Pichler Str. Nr. 30 Weiz 8169 Austria; ^2^ Johannes Kepler University Linz Dept. Physical Chemistry Linz Institute for Organic Solar Cells (LIOS) Altenberger Str. Nr. 69 Linz 4040 Austria; ^3^ Chair of Physics Department of Physics Mechanics and Electrical Engineering Montanuniversität Leoben Franz Josef Str. 18 Leoben 8700 Austria; ^4^ Institut for Chemical Technologies of Organic Materials Johannes Kepler University Linz Altenberger Str. Nr. 69 Linz 4040 Austria; ^5^ National Institute for Laser Plasma and Radiation Physics PO Box Mg‐36, Magurele Bucharest 077125 Romania; ^6^ Johannes Kepler University Linz Center for Surface and Nanoanalytics (ZONA) Altenberger Str. 69 Linz 4040 Austria; ^7^ Laboratory of Natural Materials Technology/Wood and Paper Chemistry Åbo Akademi University Porthansgatan 3‐5, Åbo Turku 20500 Finland; ^8^ Institute of Polymer Chemistry Johannes Kepler University Linz Altenberger Str. 69 Linz 4040 Austria; ^9^ Department of Chemistry and Industrial Chemistry University of Pisa via Moruzzi 13 Pisa 56124 Italy; ^10^ Present address: Mihai Irimia‐Vladu Johannes Kepler University Linz Institute of Physical Chemistry Linz Institute for Organic Solar Cells (LIOS) Altenberger Str. Nr. 69 Linz 40040 Austria

**Keywords:** green electronics, natural dielectric material, natural resins, pine resins, sustainable electronics

## Abstract

Four pinaceae pine resins analyzed in this study: black pine, shore pine, Baltic amber, and rosin demonstrate excellent dielectric properties, outstanding film forming, and ease of processability from ethyl alcohol solutions. Their trap‐free nature allows fabrication of virtually hysteresis‐free organic field effect transistors operating in a low voltage window with excellent stability under bias stress. Such green constituents represent an excellent choice of materials for applications targeting biocompatibility and biodegradability of electronics and sensors, within the overall effort of sustainable electronics development and environmental friendliness.

## Graphical abstract

Four pinaceae pine natural resins are proven to be very good dielectric materials with outstanding film forming, high breakdown field, and low interface trap density. They are processed from the green solvent ethanol and implemented as dielectrics in organic field effect transistors operating at low voltage in the frame of sustainable electronics development.

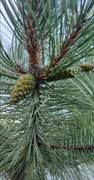

## 1 Introduction

The bioelectronics research, based on functional materials displaying biocompatibility, biodegradability and minimal toxicity toward humans and environment, has attracted recently increased interest from the scientific community as it is perceived as an alternative route to both inorganic‐ and organic‐based electronics. Bioelectronics allows reaching unprecedented functionalities of electronics targeting interaction with living tissue.^[^ ^1^, ^2^, ^3^, ^4^, ^5^, ^6^, ^7^, ^8^, ^9^ ^]^ The advent of bio‐organic electronics had a revolutionary impact on the field of organic electronics per se, by complementing it not only with additional bio integration functionalities,^[^ ^10^, ^11^, ^12^, ^13^, ^14^, ^15^, ^16^, ^17^ ^]^ but also with improved mechanical properties,^[^ ^18^, ^19^, ^20^, ^21^, ^22^ ^]^ power source abilities,^[^ ^23^, ^24^, ^25^, ^26^, ^27^ ^]^ light emitting capabilities,^[^ ^28^, ^29^ ^]^ enhanced electrical properties,^[^ ^30^, ^31^ ^]^ bio‐robotics features,^[^ ^32^ ^]^ and even harvesting the ion or proton transporting possibility.^[^ ^33^, ^34^, ^35^, ^36^, ^37^, ^38^, ^39^ ^]^ Bioelectronics is a stand‐alone area of research which is part of “*green electronics*” development, a larger umbrella considering issues of sustainability in production (low energy consumption),^[^ ^40^, ^41^, ^42^, ^43^, ^44^ ^]^ biodegradation in a controlled manner ^[^ ^45^, ^46^, ^47^ ^]^ and minimal toxicity to the environment^[^ ^48^, ^49^ ^]^ for all electronic components. Nowadays organic bioelectronics is thought to merge electronics with biological systems and even realize devices mimicking biological functions.^[^ ^50^, ^51^, ^52^ ^]^ Since research in organic materials for electronics is largely driven by the demand for restraints in costs and energy imbalance, the ease of processing is alongside the material selection an essential requisite.^[^ ^53^, ^54^, ^55^ ^]^ Nevertheless, in spite of the intensive efforts made by the scientific community, performances and reliability of organic electronic devices in general and bio‐organic electronics in particular still represent important issues and barriers to their competitiveness in comparison to their inorganic counterparts.^[^ ^56^, ^57^ ^]^ In the meanwhile, new concepts are emerging, motivating researchers to implement nonconventional thinking in finding new application niche^[^ ^58^ ^]^ exploring novel fabrication or synthesis routes for materials and devices.^[^ ^59^, ^60^, ^61^, ^62^ ^]^ Among the main motivations pushing forward the bioelectronics research direction are the compelling ecological issues caused by the society's insatiable demand of inorganic semiconductors, metals and other nonbiodegradable solid waste on one side and the increasing demand for the usage of safe, nontoxic, biocompatible and biodegradable materials and devices for applications in biomedical field and other human‐friendly electronics (temporary communication sensors, smart packaging, and devices for point‐of‐care and personalized medicine) on the other side.^[^ ^63^, ^64^, ^65^, ^66^ ^]^ These motives are triggering the current efforts pointing to the development of low cost, large volume and use‐and‐throw away devices that require a minute energy consumption compared to process materials and devices commonly used in the production of state‐of‐the‐art electronic components.^[^ ^67^ ^]^ Indeed “*the race may not be to the swift, but rather to the cheap*.”^[^ ^67^ ^]^ Inspiration from nature is a great advantage in this framework: certainly, since nature is the oldest and surely the most reliable among all the existing energy‐efficient systems.^[^ ^68^, ^69^ ^]^

As part of the general scientific effort supporting the emerging field of bioelectronics, we investigated in this work the dielectric properties of four different plant‐origin resins originating from pine tree species, i.e., *pinus nigra*, *pinus contorta*, *rosin*, *and pinus succinifera*. Resins are broadly defined as soft solid or highly viscous substances, usually containing prepolymers with reactive groups.^[^ ^70^ ^]^ The term “*resin*,” initially coined by analogy with the natural resin *rosin*, is now used in a narrower sense to refer to thermosetting polymers. Irrespective of their origin (plant or animal), natural resins are traditionally classified into three main classes:^[^ ^71^ ^]^ hard resins, gum resins and oleoresins, the latter category including turpentines, balsams and elemis. As a general rule, hard resins are freely soluble in alcohol and have a limited solubility in water and have the terpene compounds as their major ingredients. Hard resins are distinct to gums that are also tree exudates, but composed mostly of various polysaccharides and are freely soluble in water. Gum resins are mixture of gums and resins containing small amount of essential oils and are in general soluble in both water and alcohol. Gum resins are mainly produced by plant species in dry regions; gummosis can occur when resins are produced by injuries, so that carbohydrates and amino acids are incorporated into the exudate compounds.^[^ ^70^ ^]^ Oleoresins contain a higher ratio of volatile to nonvolatile compounds than other resins; balsams contain certain esters that are aromatic (e.g., benzoic or cinnamic acids), and therefore, they are commonly used as fragrances and in traditional medicine. Most of the resins belonging to these three classes have attracted deep interest for their medicinal properties and/or have been exploited in the industrial production of varnishes and lacquers and in the preparation of incenses and perfumes. While gums decompose completely upon heating without melting, resins melt – increasing their temperature over the melting point results in a progressive distillation of volatile oils. Wood resin molecules are bound to one another through intermolecular cohesive forces, which are easily disturbed when heated or dissolved.

In this work we processed all the four resins from their solubilized form in ethanol. Although resins can be solubilized also in fatty and natural essential oils, we did not pursue these avenues. The dissolved resins were filtered through a 20 µm pore hydrophilic filter and used without any further purification. They have been processed as dielectric capping layer on electrochemically grown aluminum oxide inorganic layer and dried by heating for 1 h at mild temperatures not exceeding 80 °C. The cast films were used as gate dielectric layers in p‐ and n‐type operating field effect transistors (OFETs) with pentacene and *C*_60_ semiconductors, respectively. Apart from the processability and film forming characteristics of the resins, we have investigated their composition by Gas Chromatography, their surface morphology by Atomic Force Microscopy (AFM), their surface dipole formation by Kelvin Probe Force Microscopy (KPFM), and their dielectric properties (both static and complex dielectric properties) by Impedance Spectroscopy and Ellipsometry respectively. Optical properties of the respective resins were recorded by Fourier Transform Infrared Spectroscopy (FTIR). The evolved gas analysis‐mass spectrometry (EGA‐MS) analysis was performed to investigate the thermochemistry of the resins and to highlight the occurrence of a volatile and/or macromolecular fraction within their composition. Quantitative ^31^P‐NMR spectroscopy was performed to analyze compounds in terms of mmol per gram of hydroxyl groups as phosphite derivative. Bias stress testing of the fabricated OFETs was performed in a continuous electrical stressing spanning between 12 and 14 h. The scope of this article is to bring together the field of biology, electronics fabrication and materials science for a coordinated thrust in the direction of sustainable electronics development. The article is structured in subchapters dedicated to each of the four resins considered, comprising therein the full set of investigations performed on the respective material. The summary of all the individual properties of the analyzed resins is presented in tabulated form at the end of the article text, allowing for a quick comparison of their characteristics.

## 2 Results

The results of gas chromatography measurements are comprised in **Table** **1**.

**Table 1:** Concentrations (mg/g dry ethanol extract) in species of Pine resins analyzed in this work. RA = Resin Acid; nd = not detected

Component	Black Pine	Shore Pine	Rosin	Baltic Amber
*Mono‐ and sesquiterpenoids*				
Longifolene	nd	nd	2.62	nd
Camphor	nd	nd	nd	0.941
Isoborneol	nd	nd	nd	24.1
Tetrahydrocarvone	nd	nd	nd	0.230
**Sum**	**0**	**0**	**2.62**	**25.3**
*Diterpenoids*				
RAs:				
Secodehydroabietic acids	nd	nd	nd	0.294
Pimaric acid	6.69	3.09	18.5	6.30
Sandaracopimaric acid	2.24	1.29	2.72	nd
Isopimaric acid	18.5	8.14	2.41	7.1
Abietatetraenoic acid(s)	5.73	0.818	3.37	nd
Palustric acid	7.95	4.45	1.13	nd
Dehydroabietic acid (DeAb)	64.9	25.9	38.2	5.724
Abietic acid (Ab)	17.9	7.44	29.0	nd
Abietapentaenoic acid	0.526	nd	nd	nd
Neoabietic acid	4.22	1.57	nd	nd
Dehydroabietol	nd	nd	nd	2.02
8,15‐Pimaradien‐18‐oic acid	nd	nd	nd	18.9
**Sum**	**129**	**52.7**	**95.3**	**40.32**
Oxidised RAs:				
Hydroxy‐DeAbs	32.6	8.57	8.99	nd
Hydroxy‐Ab(s)	3.64	1.91	nd	nd
Dihydroxy‐DeAb(s)	7.29	1.68	2.38	nd
Hydroxy‐7‐oxo‐DeAb	4.63	0.605	5.45	nd
Sum	**48.2**	**12.8**	**16.8**	**0**
*Other diterpenoids*:				
Thunbergene(s)	nd	0.305	nd	nd
Cis‐abienol	nd	0.434	nd	nd
Manool	nd	8.17	nd	nd
Manool oxide	nd	nd	nd	nd
Pimaral	0.561	0.366	nd	nd
Isopimaral	0.748	0.222	nd	nd
Pimarol	4.22	2.99	0.852	nd
Isopimarol	4.95	0.936	nd	nd
Dehydroabietol	nd	0.662	nd	nd
Sum	**10.5**	**14.1**	**0.852**	**0**
*Small‐molecular aliphatic acids*				
Lactic acid	2.71	1.90	1.31	3.12
3‐Hydroxypropanoic acid	0.335	nd	0.167	nd
Succinic acid	0.460	nd	0.281	nd
Levulinic acid	0.233	nd	nd	nd
2‐Methyl‐4‐oxopentanoic acid	0.226	nd	nd	nd
Methylsuccinic acid	0.100	nd	nd	nd
Ethyl‐/diethyl succinate	nd	nd	nd	13.1
Sum	**4.06**	**1.90**	**1.76**	**16.22**
*Fatty acids and alcohols*				
*n*‐Octanoic acid	nd	0.409	nd	nd
*n*‐Nonanoic acid	nd	0.108	nd	0.193
*n*‐Hexadecanoic acid	1.40	0.364	1.60	0.541
*n*‐Heptadecanoic acid	3.29	nd	nd	0.702
*n*‐Octadecanoic acid	nd	0.230	0.345	nd
*n*‐Hexadecanol	0.224	0.233	nd	nd
*n*‐Octadecanol	nd	0.205	nd	nd
Sum	**4.91**	**1.55**	**1.94**	**1.44**
*Aromatic compounds*				
Resorcinol	nd	0.353	nd	nd
Vanillin	0.321	2.89	nd	nd
Cinnamic acids	nd	2.08	nd	nd
4‐Hydroxy‐benzaldehyde	nd	0.168	0.385	nd
Ferulic acid	1.47	4.81	nd	nd
4‐Hydroxycinnamic acid	nd	1.05	nd	nd
Caffeic Acid	0.353	5.98	nd	nd
1‐Guaiacylglycerols	nd	0.377	nd	nd
Monomethyl pinosylvin	nd	0.742	0.359	nd
Isolariciresinol	nd	0.292	nd	nd
Secoisolariciresinol	nd	1.63	nd	nd
Lariciresinol	nd	15.9	nd	nd
Matairesinol	0.789	8.88	nd	nd
Pinoresinol	3.80	39.1	nd	nd
7′‐Oxolariciresinol (?)	nd	0.948	nd	nd
1,5,8‐Trimethyltetralin	nd	nd	nd	0.357
Sum	**6.73**	**85.2**	**0.359**	**0.357**
*Miscellaneous*				
Carbonic acid	1.18	nd	nd	
Glycerol	0.518	0.384	0.322	2.32
Cholestadiene	3.07	0.349	nd	14.7
Diacetone alcohol	1.16	1.45	nd	12.0
*N*,*N*‐diethylcarbamic acid	0.246	nd	nd	1.05
Ethyleneglycol	nd	2.29	nd	2.68
Sum	**6.17**	**4.47**	**0.322**	**32.7**
Identified, Sum	**210**	**173**	**120**	**116**
Unindentified Peaks	**43**	**28**	**83**	**66**
% GC Eluting	**25.3**	**20.1**	**20.3**	**18.2**

**Tables** **2** and **3** present the full list of identified compounds by high‐performance liquid chromatography‐high resolution mass spectrometry (HPLC‐HRMS) and size exclusion chromatography (SEC).

**Table 2:** HPLC‐HRMS analysis of the soluble fraction of the resins

*t*_R_ (UV) [min^−1^]	MH^+^	Name/sum formula	Rosin	Black Pine	Shore pine	Baltic amber
			*% area (UV)*			
6.15	181.0493	C_9_H_8_O_4_			10.81	
8.17	165.0544	Hydroxycinnamic acid C_9_H_8_O_3_			5.48	
9.24	195.065	C_10_H_10_O_4_		0.45	24.44	
9.93	377.1593	C_20_H_24_O_7_				
11.47	375.1438	C_20_H_22_O_7_				
11.58	327.1588	C_20_H_22_O_4_				
11.92	219.1014	C_13_H_14_O_3_				
12.39	359.1486	C_20_H_22_O_6_				
12.54	305.1380	C_17_H_20_O_5_				1
13.1	341.1382	C_20_H_20_O_5_	0.28	0.37	4.44	
13.48	333.2058	C_20_H_28_O_4_	2.17	0.94		
13.52	417.1542	C_22_H_24_O_8_				
13.75	359.1485	C_20_H_22_O_6_			8	
14.32	343.1536	C_20_H_22_O_5_				
14.62	523.196	C_29_H_30_O_9_				
14.83	317.2109	C_20_H_28_O_3_	3.61			
14.9	315.1952	C_20_H_26_O_3_	1.55			
15.01	331.1901	C_20_H_26_O_4_	6.93		2.8	
15.34	317.2108	C_20_H_28_O_3_	3.04	0.88	2.22	
15.39	507.2011	C_29_H_30_O_8_				
15.61	333.2057	C_20_H_28_O_4_	2.98			
15.80	353.2142	Ambiguous				13.23
16.54	299.2004	C_20_H_26_O_2_	3.71	1.6	3.42	
16.88	415.2112	C_24_H_30_O_6_				0.89
16.95	227.1065	C_15_H_14_O_2_		5.04		
17.18	319.2265	C_20_H_30_O_3_		7.27		
17.57	259.1901	C_14_H_26_O_4_				16.57
17.75	301.2159	Retinoic acid C_20_H_28_O_2_		5.44	0.68	
17.76	317.2108	C_20_H_28_O_3_	1.49			2.03
17.94	299.2003	C_20_H_26_O_2_	1.33	0.68		2.71
18.18	315.1952	C_20_H_26_O_3_	8.95	1.33	1.3	
18.39	317.2108	C_20_H_28_O_3_	2.65			
18.41	289.252	C_20_H_32_O_2_				
18.65	335.2214	C_20_H_30_O_4_		0.61		
19.41	305.2472	C_20_H_32_O_2_		4.58		
19.74	301.2159	C_20_H_28_O_2_	2.96	4.85	2.1	6.01
20.26	301.2158	C_20_H_28_O_2_	3.45	13.1		2.99
20.62	no mass signal	Overlapping with isopimaric acid	6.87			
20.69	303.2314	Isopimaric acid	33.47	38.54	24.2	16.2
21.05	285.2211	C_20_H_28_O		1.67		
21.29	271.2418	C_20_H_30_				
21.35	289.2522	C_20_H_32_O				
21.44	273.2573	C_20_H_32_				
21.77	273.2574	C_20_H_32_				
22.23	287.2367	C_20_H_30_O			0.39	
		**Total**:	**85.44**	**87.35**	**90.28**	**61.63**

**Table 3:** Size exclusion chromatography of the resins

Resin	*M_n_* [g mol^−1^]	*M*_w_ [g mol^−1^]	*Đ*
Black pine	299	524	1.8
Shore pine	346	841	2.4
Rosin	298	459	1.5
Baltic amber	410	1850	4.5

**Figure 1:**
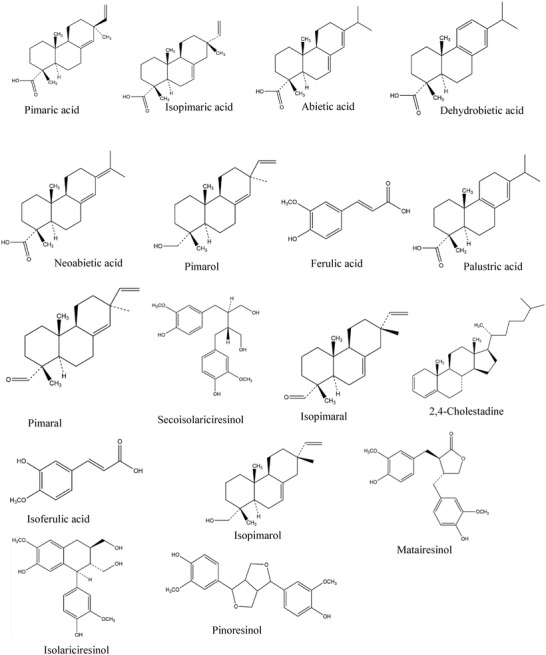
Schematic of the major constituents of the analyzed Pinaceae pine resins in this study.

The chemical structure of the most prominent constituents is presented in the schematic of **Figure** **1**.

**Figure 2:**
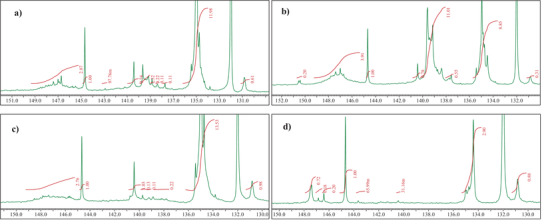
^31^P‐NMR spectra: a) Black pine; b) Shore pine; c) Rosin; d) Baltic amber.

^31^P NMR spectra are reported in **Figure** **2**a–d where the respective panels display the results in the spectral range from 130 to 150 ppm. The calculated hydroxyl content is reported as mmol per gram of sample in **Table** **4**. Integration is referred to as cholesterol, used as an internal standard.

**Table 4:** Quantitative ^31^P‐NMR analysis of hydroxyl groups reported as mmol per gram of resin. Integration limits

Chemical Group	Black Pine	Shore Pine	Rosin	Baltic Amber
Aliphatic OH^a)^	0.61	0.84	0.64	0.23
Phenolic OH^b)^	0.09	0.12	0.05	nd
Substituted Phenolic OH^c)^	0.44	2.53	0.30	nd
Carboxylic acid^d)^	2.54	1.99	2.90	0.60

^a)^ 149.0–146.0 ppm;

^b)^ phenolic hydroxyl content, 138.8–137.4 ppm;

^c)^ content of phenolic hydroxyls with substitution on C5 carbon of the aromatic ring, 143.0–139 ppm;

^d)^ 136–133.6 ppm; nd: not detectable.

This spectroscopic analysis is particularly useful for the quantification of carboxyl units present on molecular backbones, as these groups are potentially ionizable once resins are deposited in thin film. Furthermore, the analysis allows to distinguish phenol units, which are typically derivatives of shikimic acid (nonterpenic derivatives). However, the analysis records only phenol rings with a free ‐OH group, not considering the ones that underwent condensation (ether formation), which is a clear indication of molecular aging. Valuably, in these experiments, the derivatization yielded in every case clear solution, so the analyses are representative of all samples. Confirming data of GC–MS, a higher number of aromatic rings with free phenolic hydroxyls was detected in Shore Pine resin. In the other resins, their content was appreciably lower. They could not be detected in Baltic amber resin, while in black pine and rosin resins the content of aromatic rings with substituted phenolic groups was significantly lower than in shore pine resin. Baltic amber is a fossilized resin, therefore the complete lack of free phenolic groups is taken as an indication of a higher degree of condensation. This analysis confirms also the higher concentration of carboxyl groups and aliphatic alcohols in black pine and rosin resins, in agreement with results from GC–MS analyses.

### 2.1 Black Pine (*Pinus Nigra*)

#### 2.1.1 Resin Type and Composition

**Figure 3:**
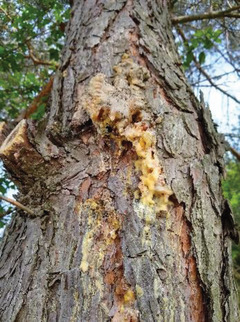
Photograph of the collected black pine resin.

The black pine (*pinus nigra*) resin was collected from a living tree growing in the city of Weiz, Austria, at an altitude of ≈480 m. The original resin deposit was captured in the photograph of **Figure** **3**. The amount of resin produced by the respective tree was really impressive, as is clearly visible in the photograph, and most likely resulted because of wounds inflicted to the tree by the city gardeners’ personnel. What is not clear though at this time is how old was the resin and also if this fact matters or not for the electronics development.

Black pine (*pinus nigra*) is a fast‐growing coniferous evergreen tree that is found in the European Mediterranean regions, the high mountains of Northwest Africa, and Asia Minor. The coverage of the black pines in the Black, Marmara and Aegean Seas, Taurus Mountain and Central Anatolia regions was estimated to be about 4.2 million ha.^[^ ^72^ ^]^ The tree was also introduced to North America by the European colonists and become soon naturalized. Black pine has two main subspecies, *pinus nigra* subsp. *salzmannii* (Corsican pine) and *pinus nigra* subsp. *nigra*, the latter also called Austrian pine, the one that provided in fact the collected resin analyzed in this work (Figure 3). Black pine trees can grow up to 40 meters high with a long‐life span of 400 years or even more. It is monoecious with the difference in color of male and female species, i.e., yellow for male catkins and reddish for female inflorescences, respectively. Ascribed to its tolerance to pollution and striking visual appearance, black pine is often planted in the parks and other plantation areas for décor purposes. The black pine wood is also widely used for general construction, fuelwood, and papermaking. Its wood finds particular use as interior floors due to its high durability, high resin content and ease of processability. For example, resin content in the heartwood of Corsican black pine is found to be as high as 20%. Turpentine can be derived from pine resin and finds numerous ethnomedicinal usages such as treatments for skin conditions, asthma, wounds, bronchitis, the common cold and cough ascribed to its strong antioxidant and analgesic effects.^[^ ^73^ ^]^ For that purpose, black tar (derived from the black pine) is still used in folk medicine, particularly in Turkey.^[^ ^74^ ^]^ The callus resin's original German term “‘*Überwallungsharz*”’ was originated to specifically describe the resin generated surrounding the wound closure of Austrian black pine. As in most pinaceae resins, resin acids were the dominating compounds in the composition of black pine, with dehydroabietic dominating (6.5% in our analysis, see data presented in Table 1 and **2**). Other important resin acids were hydroxylated dehydroabietic acids (3.3%), followed by isopimaric and abietic acid (1.8% each), palustric acid (0.79%), pimaric acid (0.67%), abietatetraenoic acid (0.57%), and neoabietic acid (0.42%). Other diterpenoids detected were isopimarol (0.49%), pimarol (0.42%), isopimaral (0.075%), and pimaral (0.056%). The diterpenoids accounted altogether for 19% of the resin weight. Other important compounds detected were the lignans pinoresinol (0.38%) and matairesinol (0.079%), ferulic acids (0.15%), and cholestadiene (0.31%). Only 25% of the material eluted from the GC column. The results of GC–MS analysis are presented in Table 1 whereas Tables 2 and **3** present the full list of identified compounds by high‐performance liquid chromatography (HPLC) and size exclusion chromatography (SEC) methods respectively. The chemical structures of the most prominent constituents of the black pine resins are presented in Figure 1.

#### 2.1.2 Structural Characterization

**Figure 4:**
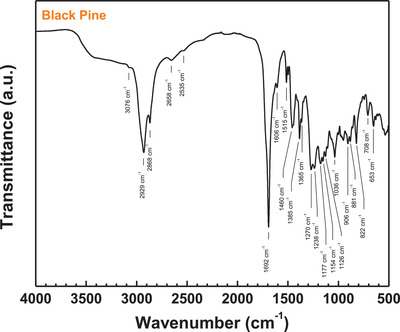
ATR‐FTIR spectrum of pine resin.

The ATR‐FTIR spectrum of black pine resin is shown in **Figure** **4**. The presence of a large share of resin acids in the material is indicated by the very broad absorption band in the wavenumber region of 3600–2500 cm^−1^, centered at around 3000 cm^−1^, which is characteristic for the O–H stretch vibration of intermolecularly hydrogen‐bonded –COOH groups. The strong C ═ O stretch vibration band at 1692 cm^−1^ further evidences the prevalence of carboxylic acid‐containing compounds. The spectral position of the band is typical for solid diterpenoid resin acids.^[^ ^75^, ^76^, ^77^ ^]^ Additionally, low intensity bands at 2658 and 2535 cm^−1^ can be observed which correspond to overtone bands of the carboxylic acid vibrations.^[^ ^75^ ^]^ The absorption bands at wavenumbers of 2929 and 2868 cm^−1^ correspond to the C–H stretching vibrations of methylene and methyl groups, while bands at 1460, 1385, and 1365 cm^−1^ stem from the bending vibrations of these groups. The band at 3076 cm^−1^ corresponds to C–H stretching vibration of unsaturated hydrocarbon molecules. Absorption bands in the wavenumber range of 1270–1036 cm^−1^ can be assigned to the O–H deformation and C–O stretching vibrations of oxygen‐containing functional groups and to the in‐plane C–H bending vibrations in unsaturated cyclic hydrocarbons, respectively. The two absorption bands at 1606 and 1515 cm^−1^ correspond to aromatic ring C = C stretching vibrations. Two strong bands at or near these two wavenumber values are typical of 4hydroxyphenyl^[^ ^78^ ^]^ or 4hydroxy3methoxyphenyl (guaiacyl)^[^ ^79^, ^80^ ^]^ substituent groups as found in lignan resin components such as pinoresinol. The low relative intensity of the absorption bands at 1606 and 1515 cm^−1^ observed for the investigated black pine resin corroborates the low fraction of phenolic lignan compounds (mainly pinoresinol) as found by GC–MS analysis. The aromatic diterpenoid dehydroabietic acid which was identified as one of the major components in the GC eluents of the resin shows no distinct C ═ C bands in this wavenumber region according to the FTIR spectrum reported for the pristine material.^[^ ^77^, ^81^ ^]^ The absorption bands in the low wavenumber region of 906–653 cm^−1^ can be assigned to the out‐of‐plane C–H bending vibrations of alkenes with different substitution patterns (e.g., vinyl groups as in isopimaric acid or trisubstituted C ═ C bonds as in abietic acid) and of aromatic rings, respectively.^[^ ^77^ ^]^ The pronounced absorption band at 822 cm^−1^ can be assigned to the out‐of‐plane C–H bending in aromatic rings and, due to the band position, may be attributed to the large fraction of dehydroabietic acid in the resin.^[^ ^77^, ^81^, ^82^ ^]^

Absorption bands in the wavenumber range of 1270–1036 cm^–1^ can be assigned to the C–O stretching vibrations of *oxygenated hydrocarbon* groups and to the in‐plane C–H bending vibrations in cyclic alkenes or aromatics (Figure 4). The absorption bands at 906, 881, 708, and 653 cm^−1^ are assigned to the out‐of‐plane C–H bending vibrations of alkenes with different substitution patterns (Figure 4) and can be related to aliphatic terpenoid resin constituents with C ═ C bonds in different positions. The absorption band at 822 cm^−1^ arises from the out‐of‐plane C–H bending in aromatic rings (Figure 4).

#### 2.1.3 Thermogravimetric Analysis

**Figure 5:**
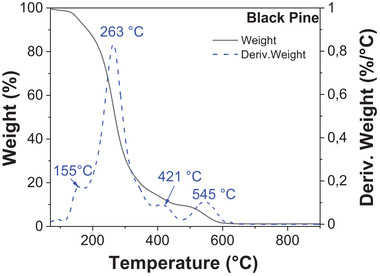
Thermogravimetric analysis of black pine.

Thermogravimetric analysis of black pine revealed exothermic reactions that start to occur when heating the resin above 240 °C (decomposition temperature, *T*_d_), under a nitrogen atmosphere (see **Figure** **5**), which is in agreement to other literature reports.^[^ ^83^, ^84^ ^]^ In the case of black pine, the first weight loss occurs below 120 °C, due to the evaporation of the adsorbed water (≈1%) and other fragrance species. The second and most pronounced weight loss ≈88%) appeared between 120 and 450 °C. The third weight loss occurs at higher temperatures, between 450 and 650 °C, where ≈9% of mass is lost. In general, three main weight loss stages or degradation zones can be attributed to hemicelluloses, 250–300 °C, cellulose, 300–350 °C, and lignin, 350–500 °C).^[^ ^85^, ^86^ ^]^ Among these temperature ranges, the weight loss from 330 to 430 °C, could be attributed to the “plasticization” transition, and precarbon formation, as reported elsewhere.^[^ ^87^ ^]^ The remaining weight of the black pine resin after the burning process at 900 °C is ≈1.12%.

#### 2.1.4 Surface Investigation

We performed surface investigation of black pine resin via contact angle measurement, AFM and KPFM.

**Figure 6:**
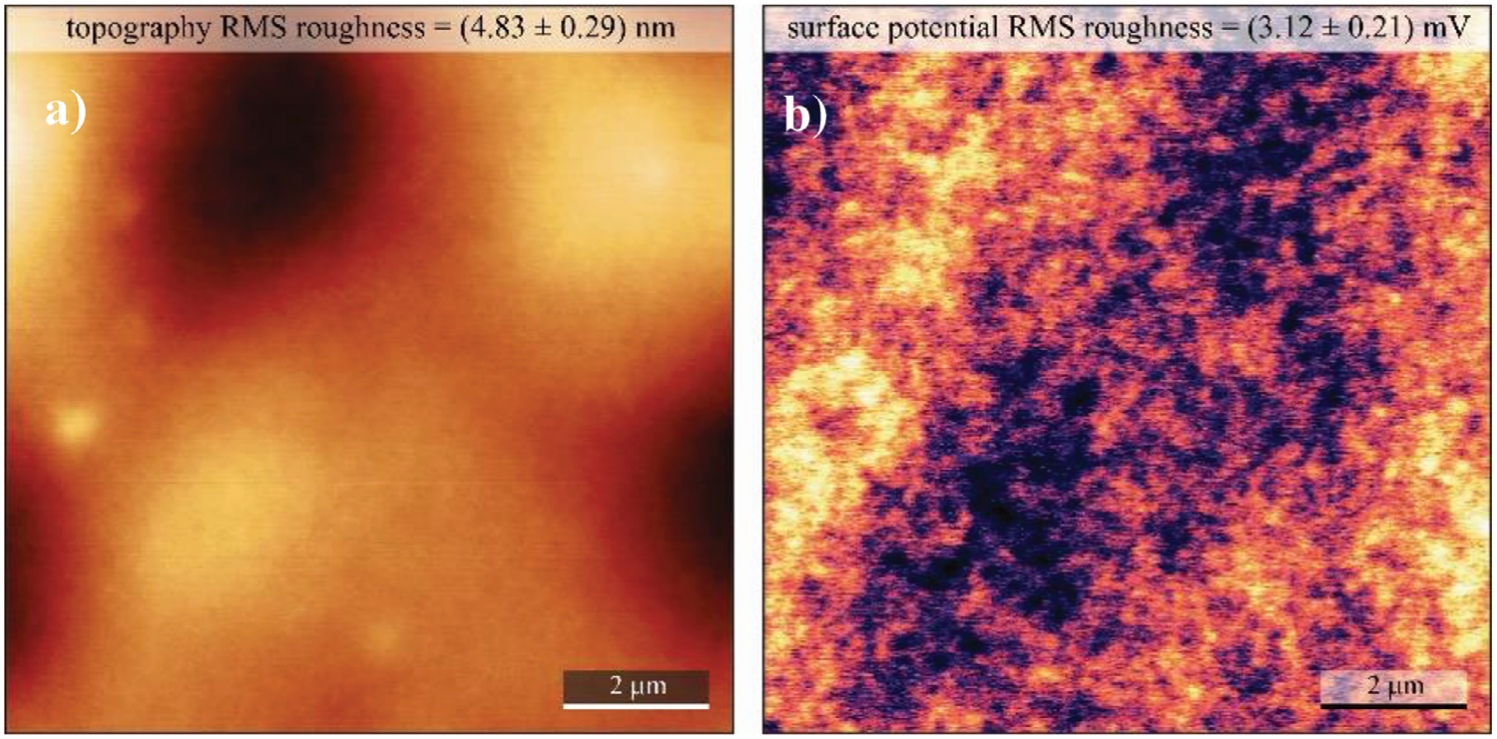
AFM and KPFM of black pine resin on gold‐coated glass. a) 10 × 10 µm^2^ topography image of the investigated surface (*z* scale 30 nm); b) corresponding surface potential map at the tip lift of 25 nm (*z* scale 20 mV). RMS roughness parameters are indicated for the corresponding images.

**Figure** **6**a presents a typical surface morphology observed for the black pine samples. The RMS roughness parameter was used to characterize smoothness of the investigated resin surfaces. RMS roughness of spin‐coated black pine surface was determined to be (4.83 ± 0.29) nm. The corresponding surface potential map is shown in Figure 6b. The main features observed in the KPFM image are correlated to higher and lower regions of the resin layer and are most likely originating simply from the probe being closer and further from the grounded electrode underneath the resin layer. The CPD RMS fluctuation was found to be (3.12 ± 0.29) mV. In comparison, clean SiO_2_ surface grown by wet oxidation of Si wafer (as a common gate insulator) measured under the same conditions yields a CPD RMS fluctuation of (17.40 ± 4.30) mV, while the surface of SiO_2_ is about one order of magnitude smoother than that of black pine resin. Far smoother surface potential map of black pine in comparison to SiO_2_ indicates lower amount of trapped charges and dipoles in the dielectric and on the dielectrics surface. These findings support the virtually hysteresis‐free behavior of the black pine‐based OFETs (to be shown in the electrical measurement section of this article), and point out to the high quality of the dielectric layer and its uniformity on the micrometer‐scale.

**Figure 7:**
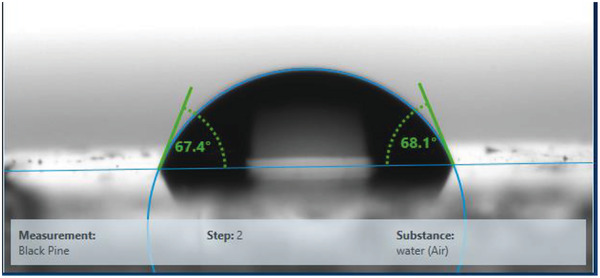
Contact angle of black pine resin. Left angle (water): 67.4°; Right angle (water): 68.1°. Total surface energy, 49.1 mN m^−1^ with a polar component of 6.9 mN m^−1^ and disperse component of 42.2 mN m^−1^.

The contact angle measurement for black pine revealed a weakly hydrophilic surface with a water contact angle of ≈68°, having a total surface energy of 49.1 mN m^−1^, separated into a disperse and a polar component of the values 42.2 and 6.9 mN m^−1^ respectively (see **Figure** **7**).

**Figure 8:**
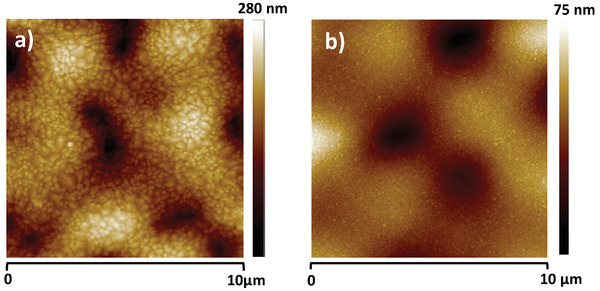
AFM scan of a) pentacene on black pine resin, RMS ≈ 33 nm and b) fullerene *C*_60_ grown on black pine resin, RMS ≈ 7 nm. The two scans were performed in the channel of the measured OFET devices presented in Figure 10.

The topography of the two semiconductors grown on pine resin is presented in **Figure** **8**. The morphology of the pentacene and *C*_60_ differs significantly, as Figure 8 shows. Both grains are uniform in size, but pentacene grows in aggregates of grains of typical size of 200 to 300 nm and an RMS surface roughness of ≈33 nm, whereas the grains of *C*_60_ are very small, in the range of 10 to 20 nm, with an RMS surface roughness of ≈7 nm.

#### 2.1.5 Dielectric Investigation

Impedance spectroscopy represents a very informative investigation for understanding the processes that occur at interfaces between two different materials, e.g., processes that lead to changes in physical properties of the system, i.e., electrical, crystallographic, mechanical or even compositional.^[^ ^88^ ^]^ In the present case, impedance spectroscopy helps explaining changes in electrical properties of the system by studying the influence of polarization on the variation of electrical conductivity of the dielectric film. Thus, by performing the conductivity measurement over a wide range of frequencies (i.e., 1 MHz to 1 mHz), impedance (dielectric) spectroscopy offers valuable pieces of information on various conductive species and pathways, each of them being active in a particular frequency window. Here, we were interested to see possible relaxation of the loss angle and sharp increase of capacitance at low frequencies (below 1 Hz), both correlated events indicating the presence of mobile ionic species in the dielectric.^[^ ^89^, ^90^, ^91^ ^]^

**Figure 9:**
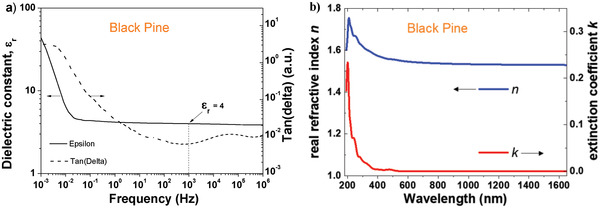
a) Impedance measurement of black pine resin; b) complex refractive index of black pine resin.

We performed dielectric measurements on a 218 nm thick film of black pine resin cast from a 0.1 g mL^−1^ stock solution in ethanol, spin coated and dried on 1 mm wide aluminum electrode in a metal–insulator–metal configuration, with aluminum as the top electrode material. We measured the dielectric spectroscopy between 1 MHz and 1 mHz and observed a very uniform capacitance all over the measurement range down to about 30 mHz, followed by an increase by almost one order of magnitude. The respective frequency window (i.e., 1 to ≈30 mHz, see **Figure** **9**) is not relevant for solid state electronics and indeed the OFETs with black pine dielectric showed minimal hysteresis in both transfer and output characteristics, as it will be shown in the transistor measurement section.

From the plotted data of the capacitance at 1 kHz, we extracted a dielectric constant of 4 for black pine resin. The spread of the dielectric constant for the 16 investigated MIM structures was ±0.1 for black pine. The film of 218 nm thick given here as an example broke at 124 V, which translates into a breakdown field of 5.7 MV cm^−1^ for black pine resin processed from ethanol. All the 16 analyzed MIM structures consisted of resin films cast from the same solution and at an identical rotation speed, therefore the variation of film thickness was consequently minimal. We did not pursue a classic study of breakdown field that takes into consideration the variation of the thickness of the dielectric in order to establish a Weibull distribution of the results, and this will be discussed further in the Section 4 of this article.

The isotropic complex refractive index, *n* and *k*, of black pine is displayed in Figure 9b). Black pine has multiple absorption bands within the UV tailing out into the visible range, which gives the resin a slightly colored appearance in thicker layers. Otherwise, the real refractive index n has little dispersion ranging between 1.6 and 1.5 being similar to silicon dioxide or glass.

#### 2.1.6 Electrical Measurements

**Figure 10:**
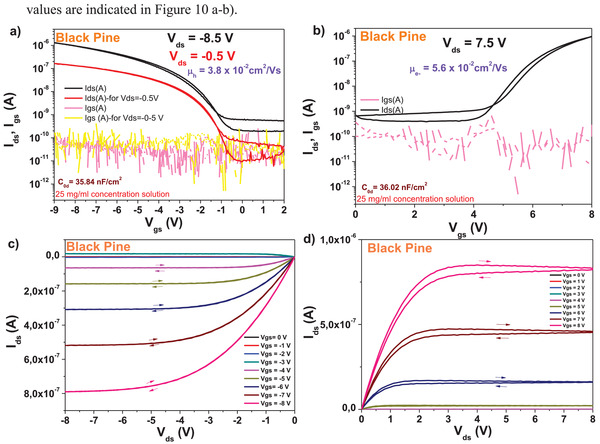
Transistor measurements of black pine resin on AlOx gate with pentacene and *C*_60_ as organic semiconductors. a,b) are the transfer characteristics at different drain voltages *V*_ds_ and c,d) the output characteristics for gate voltages 0 ≤ *V*_gs_ ≤ 9 V of pentacene and 0 ≤ *V*_gs_ ≤ 8 V for *C*_60_ OFETs, respectively. The capacitance *C*_0d_ is shown as inset in panel a) for pentacene and in panel b) for *C*_60_. Also indicated are the hole and electron mobilities, µ_h_ and µ_e_ respectively, as well as the concentration of precursor resins solutions in ethanol.

We fabricated field effect transistors on black pine resin capped aluminum oxide gate electrode, with both pentacene and *C*_60_ semiconductors. The source and drain electrodes of the devices were made from aluminum in case of fullerene and gold in case of pentacene. The OFET devices were fabricated with the organic semiconductors (i.e., pentacene and *C*_60_) deposited on top of a combo dielectric layer comprised of electrochemically grown aluminum oxide inorganic layer (anodized to 10 V, having a thickness of ≈18 nm) capped by a thin layer of black pine resin cast from a 25 mg mL^−1^ stock solution and dried at 80 C for 1 hour in air on top of a hot plate. The typical thickness of the organic resin dielectric was in the range of 60 nm, and the specific capacitance *C*_0d_ of the bilayer was situated in the range of 36 nF cm^2^. The devices specific *C*_0d_ values are indicated in **Figure** **10**a,b).

The typical transistor characteristics of pine resin dielectric showed hysteresis free behavior both in transfer and output characteristics for pentacene semiconductor (see Figure 10a), but at the same time it displayed a significant hysteresis for the fullerene semiconductor especially in the output characteristics (see Figure 10,d). The dielectric behavior was characterized by low leakage in the range of 100 pA all throughout the measurement range (i.e., 0–8 V for fullerene semiconductor and 2 to −9 V for pentacene semiconductor). The calculated semiconductor mobility was in the range of 0.03 to 0.05 cm^2^ V^−1^ s^−1^ for both type of semiconductors involved in the study. As explained in the Experimental Section, we did not pursue in this work the avenue of obtaining record mobilities for the organic semiconductors, but investigated the dielectric behavior instead. With this respect, we did not optimize the deposition of the organic semiconductors to the particular interface (each resin), but used a standard, identical recipe for all the pine resins. Moreover, we did not employ in this study an organic semiconductor of ultimate purity (but merely a one‐time grade purified by sublimation), a fact that could have had a significant influence over the final mobility values, as thoroughly presented in our recent study.^[^ ^92^ ^]^ The subthreshold swing of the two semiconductors deposited on black pine capping layer of AlOx dielectric was 0.9 V dec^−1^. for pentacene and 0.8 V dec^−1^. for fullerene. In the same time, the normalized subthreshold swing values were 17.1 V nF cm^−2^ dec^−1^. for pentacene and 28.7 V nF cm^−2^ dec^−1^. for *C*_60_. As it was the case with the calculated field effect mobility, also the normalized subthreshold swing was in the same value range for both pentacene and fullerene transistors, despite the slight difference in the thickness of the black pine dielectric, which is visible in the small discrepancy of the normalized capacitances, *C*_0d_. A characteristic of black pine resin was the inducing of relatively high OFF level of the organic transistor characteristics (a fact more prominent in the case of fullerene), possibly because of the tendency of the dielectric material to charge the semiconductor in its OFF state.

**Figure 11:**
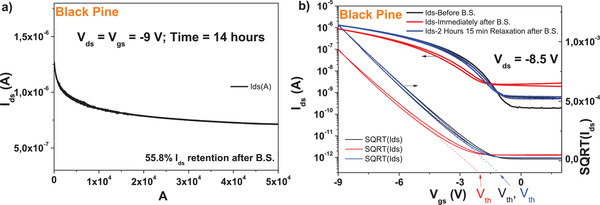
a) Bias stress (B.S.) and b) recovery after B.S. of the black pine OFET transistor characteristics with pentacene semiconductor. Scans were performed at 15 min interval, but the rest of the curves were omitted from the graph to avoid cluttering.

We performed bias stress measurement of the pentacene‐based OFET used for transfer and output characteristics, and the results are shown in (a) and (b). We stressed the device at the maximum voltage used for transfer measurement (i.e., −9 V), while keeping both these drain and gate voltages constant for 14 h stress time. We measured the transfer characteristic at the beginning of the test, as well as immediately after releasing the electrical stress, and recorded a ≈56% retention of the I_ds_ current after releasing the bias stress. We continued to measure the recovery curve of the devices with 5 min increment, but for the simplicity and the avoidance of the cluttering of the graph, we show only the transfer curve where the full or nearly full recovery was measured. In the case of black pine, the ON level of the drain current, as well as the threshold voltage, recovered after 2 h and 15 min relaxation after the bias stress time of 14 h. A similar bias‐stress measurement with *C*_60_ semiconductor provided more modest results (data not shown), with below 15% I_ds_ retention after completion of the bias stress, and recovery in ≈6 h (**Figure** **11**).

**Figure 12:**
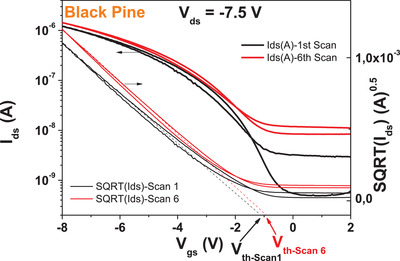
Consecutive measurement stability for black pine resin dielectric with pentacene semiconductor. The *V*_th1_ and *V*_th6_ are the threshold voltages for the 1st and 6th consecutive scan respectively.

We performed also the brief stability under consecutive scanning experiment for black pine‐based OFETs with pentacene semiconductor (see **Figure** **12**), and observed that black pine resin and pentacene OFET behaves rather modest regarding this type of measurement stability, with about 200 mV shift of the threshold voltage after only 6 consecutive scans. In the same time the transfer characteristics showed a significant (one order of magnitude increase of the OFF level) of the device, alongside the substantial (≈18%) increase in ON current, from 1.21 × 10^−6^ to 1.42 × 10^−6^ A (see Figure 12).

### 2.2 Shore Pine (*Pinus Contorta*)

#### 2.2.1 Resin Type and Composition

**Figure 13:**
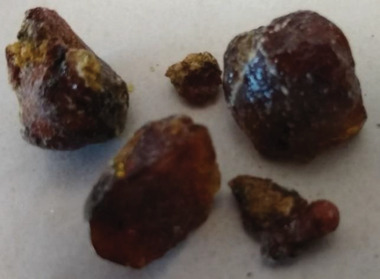
Photograph of the shore pine resin.

The samples of *pinus contorta* were collected in August 2017 near Buffalo hump, Nez Perce National Forest, Northern Idaho, USA, at an altitude of circa 2500 m. We did not record, unfortunately a picture of the resin in its host tree, but are able to present instead, the picture of the collected resins in **Figure** **13**. *Pinus contorta* is an evergreen coniferous, who grows on the coastal areas or mountain slopes of the North American continent, and is praised for both its high wood quality as well as its exquisite ornamental purposes.^[^ ^93^ ^]^ *Pinus contorta* has quite a few subspecies,^[^ ^94^ ^]^ and depending on the respective subspecies, it can be broadly divided into shrub and tree lines. The shrubs can grow up to 3 m tall, while trees can reach heights of 50 m,^[^ ^95^ ^]^ with the tallest subspecies known being *murrayana*. Shore pine occasionally reaches 300 years of age, but in reality, rarely lives to become that old. The reason behind it is that when pinus contorta reaches full maturity (i.e., about 100 years of age), it is attacked by bark beetles, particularly the mountain pine beetle, for which the tree does not have adequate defense mechanisms.^[^ ^96^ ^]^ In a recent study of Martinson et al. about beetle attack on *pinus contorta* it was showed that slower growing trees produced less resin than faster growing conspecifics^[^ ^97^ ^]^ and therefore are more prone to the attack of the beetle due to limited resin production to help in defense; the resin is believed to engulf the larvae of the beetle and suppress their development to adult beetles, therefore defending the tree in the process. The resin of *pinus contorta* was exploited for its medicinal uses by the natives of North America: as an ointment for relieving the rheumatic pains or other sores, or as a chewing gum to treat sore throats.^[^ ^98^ ^]^

In this resin, as demonstrated by our GC analysis, lignans formed the dominating compound group (8.5%), followed by diterpenoids (8.0%). The dominating compound was pinoresinol (3.9%), followed by dehydroabietic acid (2.6%) and lariciresinol (1.6%). Other important detected compounds were the resin acids isopimaric acid (0.81%), abietic acid (0.74%), palustric acid (0.44%), pimaric acid (0.31%), and neoabietic acid (0.16%) and the other diterpenoids pimarol (0.30%), isopimarol (0.094%), pimaral (0.037%), and isopimaral (0.022%). Important detected aromatic compounds were matairesinol (0.89%), caffeic acid (0.60%), ferulic acids (0.48%), vanillin (0.29%), cinnamic acids (0.21%), secoisolariciresinol (0.16%), and isolariciresinol (0.029%). Cholestadiene accounted for 0.035% of the resin weight. Only 20% of the material eluted from the GC column. Although the two pine trees (*pinus nigra* and *pinus contorta*) may carry some physical resemblance to one another, the chemical content and composition of their resins differed substantially, as demonstrated by our composition analysis. In addition to GC analysis, we also performed high‐performance liquid chromatography (HPLC) and size exclusion chromatography for the shore pine resin, and the findings are presented in Tables 2 and 3, respectively. The chemical structure of the most prominent constituents of the shore pine resin is presented in Figure 1.

#### 2.2.2 Structural Characterization

**Figure 14:**
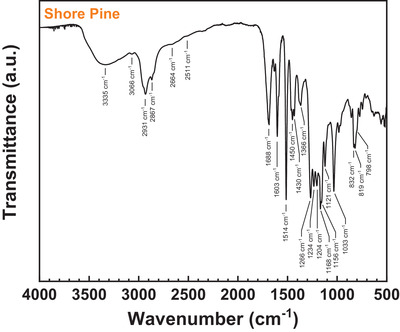
ATR‐FTIR spectrum of shore pine.

**Figure** **14** shows the ATR‐FTIR spectrum measured for the investigated shore pine resin. The spectrum features a broad absorption band centered at 3335 cm^−1^ which is characteristic of a hydroxyl group O–H stretching vibration broadened by hydrogen bonding. The band can be attributed to the large share of phenolic lignan compounds in the resin which comprise predominantly pinoresinol and lariciresinol. The large fraction of phenolic lignans in the resin as found by GC–MS analysis is further corroborated by the large relative intensity of the characteristic aromatic C ═ C stretching vibration bands at wavenumbers of 1603 and 1514 cm^−1^ .^[^ ^78^, ^79^, ^80^ ^]^ The FTIR spectrum also shows a very broad carboxylic acid O–H stretching band in the region of ≈3600–2500 cm^−1^ which is superimposed by the hydroxyl OH and the unsaturated hydrocarbon C‐H (3066 cm^−1^) and saturated hydrocarbon C–H stretching bands (2931 and 2867 cm^−1^). Other characteristic carboxylic acid bands are the overtone bands at 2664 cm^−1^ and 2511 cm^−1^ and the C ═ O stretching vibration band at 1688 cm^−1^. The considerably lower relative intensity of the carboxylic acid bands as compared to the FTIR spectrum of black pine resin corroborates the lower share of diterpenoid acids in the shore pine resin as found by GC–MS. The absorption bands at 1450–1366 cm^−1^ can be assigned to C–H bending vibrations of –CH_2_– and –CH_3_ groups. The bands in the wavenumber range of 1266–1033 cm^−1^ can be assigned to O–H deformation, C‐O stretching vibrations and to in‐plane C–H bending vibrations, respectively. Major differences of the FTIR spectra of shore pine and black pine can also be seen in the wavenumber region below 1000 cm^−1^, where a significantly different band pattern with comparatively strong coalescing bands at 832–798 cm^−1^ can be observed for shore pine. These bands can be assigned to the out‐of‐plane C–H bending vibration of different unsaturated compounds in the resin and further corroborate the different compositions of the resins as found by GC–MS analysis.

#### 2.2.3 Thermogravimetric Analysis

**Figure 15:**
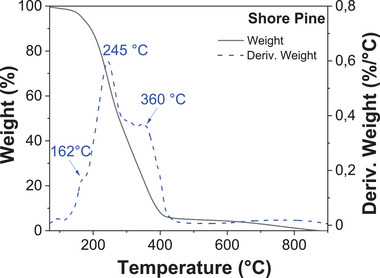
Thermogravimetric analysis (TGA) of shore pine.

The thermal stability of shore pine was analyzed by TGA using the same experimental heating setup as for black pine and all other resins. For this resin (**Figure** **15**), a first weight loss was detected below 120 °C (0.93%) corresponding to the adsorbed water. The second and larger pronounced weight loss was centered at 245 °C, were the sample lost ≈93% of its weight between 120 °C and 450 °C. In this temperature range, two other decomposition temperatures were detected, at 162 and 360 °C respectively. From 450 to 650 °C a very small weight lost was measured (1.63%), followed by a final 3.9% weight loss between 650 and 900 °C, which left a negligible residual weight of 0.48%, due to an almost whole decomposition of the resin.

#### 2.2.4 Surface Investigation

**Figure 16:**
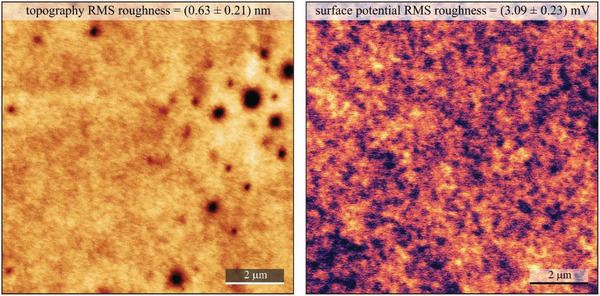
AFM and KPFM of shore pine resin on gold‐coated glass. Left panel: 10 × 10 µm^2^ topography image of the investigated surface. Right panel: corresponding surface potential map at the tip lift of 25 nm. Average RMS parameters are indicated for both, the topography roughness and the CPD fluctuations.

We performed surface investigation of shore pine resin via contact angle measurement, AFM, and KPFM. In contrast to the other resin films considered in this study, small and shallow holes (below 1 µm in diameter and only 1–2 nm deep) were observed on the surface of the shore pine resin film. These features do not penetrate through the entire film, and were also found to have no influence on the spatial variation of surface potential. RMS roughness of spin‐coated shore pine surface was determined to be (0.63 ± 0.21) nm, which is almost one order of magnitude smaller than for the black pine. Interestingly, the RMS value of CPD fluctuation was found to be (3.09 ± 0.23) mV, which is very similar to the values observed on the black pine samples, also pointing toward virtually trap‐free surface. In fact all the analyzed samples were very smooth with an RMS roughness well below 1 nm (**Figure** **16**).

**Figure 17:**
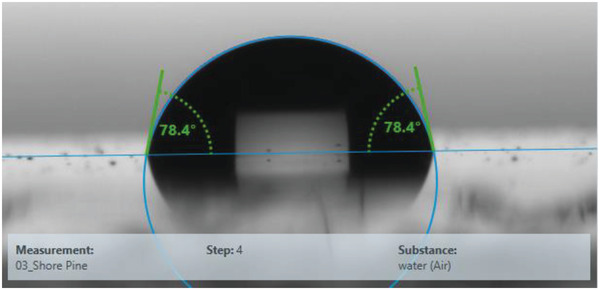
Contact angle of shore pine resin. Left angle (water): 78.4°; Right angle (water): 78.4°. Surface energy 44.0 mN m^−1^ with 40.3 mN m^−1^ as a disperse component and 3.7 mN m^−1^ as a polar component.

The measured contact angle of around 70° (**Figure** **17**) indicated a weakly hydrophilic surface for shore pine resin, similar as for black pine resin. The contact angle with water was ≈78° and the surface energy 44.0 mN m^−1^ of which 40.3 mN m^−1^ is the disperse component and 3.7 mN m^−1^ the polar component.

**Figure 18:**
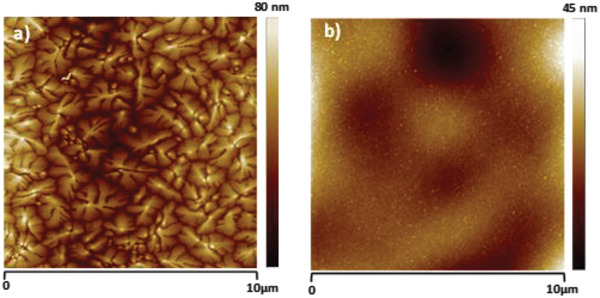
AFM scan of a) pentacene on shore pine resin, RMS ≈ 45 nm and b) fullerene *C*_60_ grown on shore pine resin, RMS ≈ 4 nm. The two scans were performed in the channel of the measured OFET devices presented in Figure 20.

The topography of the semiconductors grown on shore pine resin is presented in **Figure** **18**. Both grains are uniform in size, but pentacene grows in very large, dendritically oriented aggregates of typical size of 2–3 µm and a surface roughness, RMS ≈ 45 nm, whereas the grains of *C*_60_ are very small, in the range of 10 nm or smaller, and a surface roughness, RMS ≈ 4 nm.

#### 2.2.5 Dielectric Investigation

**Figure 19:**
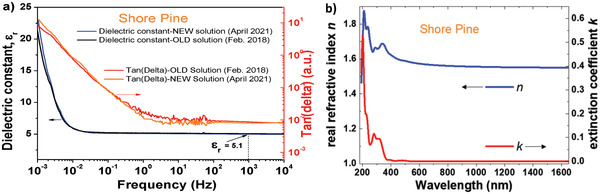
a) Impedance measurement of shore pine; b) complex refractive index *n*, *k* for shore pine.

We performed dielectric measurements on a thin film of 420 nm thick of shore pine resin spin coated and dried on 1 mm wide aluminum electrode in a metal–insulator–metal configuration, with aluminum as the top electrode material. We observed a uniform capacitance from 10 kHz to 10 mHz, but at lower frequencies (10–1 mHz) the capacitance increased abruptly as visible in **Figure** **19**. Moreover, the loss angle also increases for frequencies below 0.5 Hz thereby revealing some sort of ionic movement (see Figure 19). From the capacitance of the film at the frequency of 1 kHz, and given the thickness of the film of 420 nm, we calculated a dielectric constant of 5.1 for shore pine, with the statistical deviation of ± 0.2 for all the 16 MIM samples measured. We measured in fact the impedance spectroscopy on two different solutions stemming from the same precursor resin (same pellet): one solution produced in February 2018 and preserved in the closed vial on a laboratory shelf, and the other one produced in April 2021. We observed that the increase of both capacitance and loss angle at low frequencies is not due to the aging of the solution dated 2018, but it is an intrinsic property of the resin itself, as it is visible in the Figure 19a. The reason for the ionic movement at very low frequencies in shore pine resin film is not immediately clear. We are working to investigate the major contributor for this event. The film of 420 nm thick shore pine broke at 286 V which translates into a breakdown field of 6.8 MV cm^−1^. As it is visible in the figure of breakdown field of the four resins investigated in this work (see the final chapter), in difference to black pine, shore pine did not break fully, but rather the material ceded in one section only at 286 V applied voltage, while losing one order of magnitude from the measured capacitance (i.e., 5.6 × 10^−11^ F to 9.6 × 10^−12^ F). The dielectric was able to maintain the plateau value of the capacitance of 9.6 × 10^−12^ F for another 100 V incrementally applied with 2 V step and 2.5 s. waiting time at each applied voltage. The film finally broke and the measured capacitance moved to negative displayed values (fully broken film) at 388 V, which translates into a complete breakdown film of 9.2 MV cm^−1^, a really impressive value (see Figure 44). As in the case of the other 3 resins evaluated in this study, we did not pursue a classic investigation of the breakdown field that takes into consideration the variation of the thickness of the dielectric.

The isotropic complex refractive index, n and k, of shore pine is displayed in Figure 19b. It is very similar to black pine but has more pronounced absorption bands within the UV spectral range.

#### 2.2.6 Electrical Measurements

**Figure 20:**
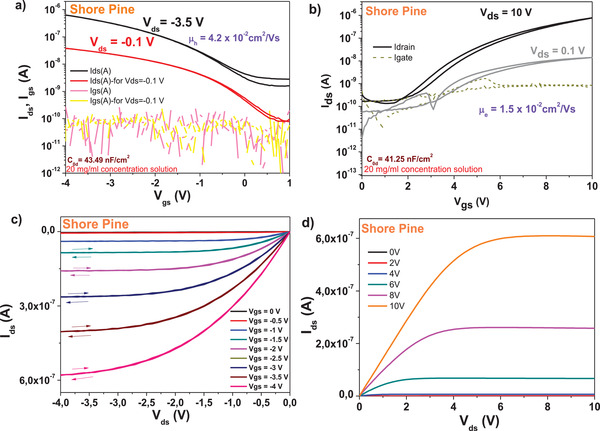
Transistor measurements of shore pine resin on AlOx gate with pentacene and *C*_60_ as organic semiconductors. a,b) are the transfer characteristics at different drain voltages *V*_ds_ and c,d) the output characteristics for gate voltages 0 ≤ *V*_gs_ ≤ 4 V of pentacene and 0 ≤ *V*_gs_ ≤ 10 V for *C*_60_ OFETs, respectively. The capacitance *C*_0d_ is noted in a) for pentacene and in b) for *C*_60_. Also indicated as insets are the hole and electron mobilities, *µ*_h_ and *µ*_e_ respectively, as well as the concentration of respective resins solutions in ethanol.

We fabricated field effect transistors on shore pine resin capped aluminum oxide gate electrode, with both pentacene and *C*_60_ semiconductors deposited on top of a combo dielectric layer. The inorganic dielectric comprised of electrochemically grown aluminum oxide inorganic layer (anodized to 10 V, having a thickness of ≈18 nm). The capped thin layer of shore pine resin was spun from a 20 mg mL^−1^ stock solution at 2500 rpm speed and dried at 80 °C for 1 h in air on top of a hot plate. The typical thickness of the organic resin dielectric was in the range of ≈50 nm, and the specific capacitance of the combo layer was situated in the range of ≈43 nF cm^−2^. The devices were finalized by source and drain electrodes made from aluminum in case of *C*_60_ and gold in case of pentacene. Transfer and output characteristics of OFET devices fabricated with shore pine resin in ethanol as capping layer spin coated on electrochemically grown aluminum oxide gate dielectric are presented in **Figure** **20**. Although the operation voltage of the two devices differ significantly, the two semiconductors field effect mobilities were comparable, i.e., 4 × 10^−2^ and 1.5 × 10^−2^ cm^2^ V^−1^ s^−1^ for pentacene and *C*_60_, respectively.

The device with fullerene showed minimal hysteresis, whereas the pentacene device was virtually hysteresis free in both the transfer and the output characteristics. In line with other resins analyzed in this group, also shore pine displayed a high OFF level in transfer characteristics with pentacene channel (i.e., in the range of 3 nA, see Figure 20a), whereas the OFF level for the fullerene channel was one order of magnitude lower (Figure 20b), with values around 0.2 nA). The subthreshold swing of the two devices was recorded in the same range as for the black pine resin, i.e., 1 V dec^−1^. for the pentacene and 1.3 V dec^−1^. for the fullerene OFET. Given the similar specific capacitance of the two types of OFETs, the normalized subthreshold swing values were in a similar range too for the two semiconductors, i.e., 43 and 53 V nF cm^−2^ dec^−1^ for pentacene and *C*_60_, respectively.

**Figure 21:**
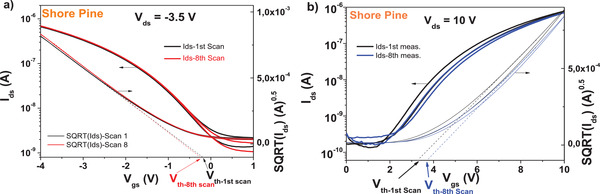
Shore pine resin as dielectric with a) pentacene and b) *C*_60_ measured for stability under consecutive scanning. In both panels, *V*_th_ stands for the threshold voltage.

We pursued a short consecutive scan test with pentacene, displayed in **Figure** **21**a) and found a very good stability of the device parameters in terms of threshold voltage (i.e., only 1 mV shift was recorded after 8 scans) as well as ON and OFF levels (where variation in the range of 1% of the initial value of the current was observed). Interestingly though, the ON–OFF ratio of the device slightly improved after 8 consecutive scans. Nevertheless, a similar device with *C*_60_ semiconductor showed a significantly inferior performance, with about 280 mV shift of the threshold voltage and a decrease of the ON–OFF ratio at the end of the consecutive scanning.

**Figure 22:**
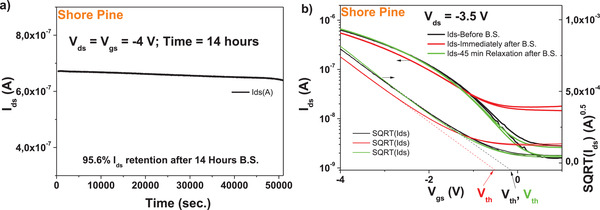
a) Bias stress and b) recovery after bias‐stress of the shore pine OFET characteristics with pentacene semiconductor.

Bias stress test was also run for a device with shore pine as capping layer on aluminum oxide dielectric and pentacene semiconductor (see **Figure** **22**a). The results showed that the device was very stable with bias stress during the initial 14 h of testing, with an *I*_ds_ retention in excess of 95%, which places the device among the most performant devices ever reported for bias stress.^[^ ^49^, ^99^, ^100^, ^101^, ^102^ ^]^ The stability after bias stress and the full recovery are presented in Figure 22b. The shore pine device fully recovered in terms of ON–OFF ratio and threshold voltage when 45 min relaxation time passed at the end of the bias stress. We want to point out that this value ranks shore pine OFET with pentacene semiconductor among the world best organic semiconductor devices with respect to recovery after bias stress.^[^ ^99^, ^103^ ^]^

A bias‐stress measurement with *C*_60_ semiconductor offered modest results (data not shown), with about 20% *I*_ds_ retention after completion of the bias stress, and recovery in ≈4 h.

### 2.3 Rosin

#### 2.3.1 Resin Type and Composition

**Figure 23:**
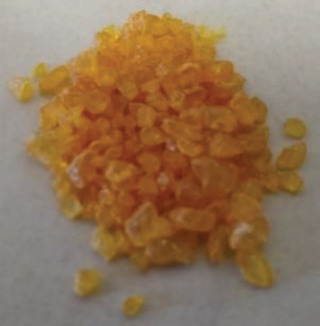
Photograph of the rosin resin. Rosin was purchased from Aldrich, product No 60 895, Lot No. BCBB0879V.

Rosin, (**Figure** **23**) also called “*colophony*,” originates from the fluid oleoresin in resinous tree species, primarily of pines of different species. The rosin can be produced either by extraction with solvent from pine stumps, wood rosin, or by processing the collected fresh oleoresin extrudes from standing trees where in the latter case gum rosin is the residue of the extraction process.^[^ ^104^ ^]^ In addition, tall oil rosin is another major rosin type and is usually obtained by distillation of crude tall oil, a side product of kraft pulping process. The rosin production volume worldwide was estimated to be 1.157 million tons in 2020^[^ ^105^ ^]^ with the share of gum rosin, 64.4%, tall oil rosin, 34.7%, and wood rosin: 0.9%. Production of gum rosin by tapping from pine trees has a long tradition. The use of such rosin as a medicine against ulcers and sores can be traced back ≈1700 years ago in China.^[^ ^106^ ^]^ Rosin mainly consists of different resin acids with smaller amounts of other diterpenoid alcohols and aldehydes.^[^ ^107^ ^]^ Rosins and their derivatives find broad applications such as adhesives and sealants, printing inks, paper size, emulsifiers, coatings, and other chemicals.^[^ ^108^ ^]^ Rosin employed in this study was purchased from Sigma‐Aldrich, product No. BCBB0879V and according to the information of the MSDS is derived especially from pine wood, composed primarily of resin acids (primarily abietic acids) and other modified resin acids. Rosin is being typically produced by heating the liquified fresh resin up to the vaporization point of volatile liquid terpene components.^[^ ^109^, ^110^ ^]^ We analyzed the composition of several other resins by gas chromatography (not included in this report, i.e., fir, larch, cedar or spruce as part co coniferous Pinaceae family, or several other resins stemming from Cupressaceae trees), and observed that rosin's composition matches indeed the one of pine resins (*pinus nigra* and *pinus contorta*) with respect to the eluting constituents. Indeed, this resin had a closer chemical content and composition (determined by GC) with the black pine resin (see Tables 1 and 2), although the exact percentage of the composition differs significantly. With this respect, the content of the dominating compound dehydroabietic acid (3.8%) was lower than in the black pine resin, and the content of unidentified GC eluting compounds was higher (8.3% compared to 4.3%). Other noticeable detected compounds were the hydroxylated resin acids (altogether 1.68%), the resin acids abietic acid (0.29%), pimaric acid (0.18%), abietatetraenoic acids (0.34%), sandaracopimaric and isopimaric acid (0.27% and 0.24%, respectively), and palustric acid (0.11%). Aromatic compounds were detected in very low amounts (altogether 0.074%), and lignans and cholestadiene were not detected at all. It is possible that decarboxylated resin acids are found among the unidentified compounds, because some of them may not be present in mass spectral databases. The resin acid dimers may be undetectable by GC, especially if they are hydroxylated, because of the high molar mass of their silylated derivatives.

#### 2.3.2 Structural Characterization

**Figure 24:**
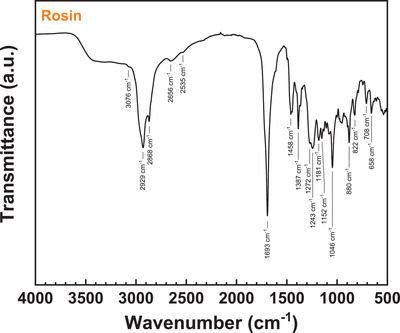
ATR‐FTIR spectrum of commercial rosin.

**Figure** **24** shows the ATR‐FTIR spectrum of the investigated commercial rosin resin. Similar to the FTIR spectrum of black pine, rosin shows very pronounced absorption bands associated with carboxylic acid vibrations. The very broad absorption band at 3600–2500 cm^−1^ corresponds to the carboxylic acid O–H stretching band, broadened by hydrogen bonding. The carboxylic acid overtone bands at 2656 and 2535 cm^−1^ and the C ═ O stretching band at 1693 cm^−1^ show identical position as in black pine resin. This corroborates the similar general compositions of the GC eluents of the two resins with diterpenoid resin acids (RA) as the clearly dominating constituents. Identical band positions and relative intensities as in black pine can also be observed for the unsaturated and saturated hydrocarbon C–H stretching vibrations at 3076, 2929, and 2868 cm^−1^, respectively, as well as the methyl and methylene group bending vibrations in the region of 1460 to 1385 cm^−1^. Differences between the FTIR spectra of rosin resin and black pine resin can be observed in the wavenumber region of 1272–1046 cm^−1^ which shows absorption bands at different spectral positions. These differences can be attributed to different fractions of the prevalent RA components in the two resins. Furthermore, the spectrum of rosin shows no distinct phenolic lignan aromatic C ═ C stretching vibration bands in the region of 1606–1515 cm^−1^ as opposed to black pine and shore pine resin. This conforms well to the results of GC–MS analysis which did not identify appreciable amounts of phenolic lignan compounds in the resin. Bands at similar positions as in black pine resin can also be observed in the low wavenumber region of the rosin FTIR spectrum at wavenumbers of 880, 822, 708, and 658 cm^−1^ which can be assigned to the out‐of‐plane C–H bending in unsaturated hydrocarbons. The low relative intensity of the band at 822 cm^−1^ as compared to black pine can be attributed to the lower share of dehydroabietic acid^[^ ^77^, ^81^ ^]^ in the rosin resin while the higher intensity of the band at 880 cm^−1^ could be attributed to a larger fraction of abietic acid.^[^ ^77^ ^]^

#### 2.3.3 Thermogravimetric Analysis

**Figure 25:**
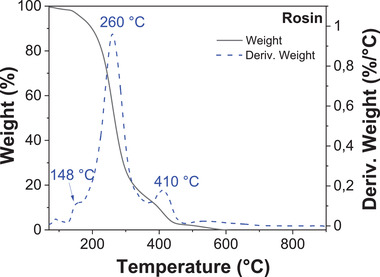
Thermogravimetric analysis of rosin.

Rosin was analyzed by TGA (see **Figure** **25**), where a first weight loss was detected below 120 °C (1.11%) due to adsorbed water and other volatile species. The second important weight loss was centered at 260 °C, where the sample lost ≈95.4% of its weight, starting at 120 °C and finishing at 450 °C. As in the case of shore pine, two other decomposition temperatures were detected, at 148 and 410 °C respectively. In the case of rosin, a weight loss of 3.0% was measured, from 450 to 650 °C. Finally, an insignificant 0.34% weight loss after 650 °C left almost no residual weight (0.06%), due to a nearly complete decomposition of the resin. This event is probably a consequence of the distillation of the commercial rosin resin, which was purified by removal of low volatile species or other impurities.

#### 2.3.4 Surface Investigation

**Figure 26:**
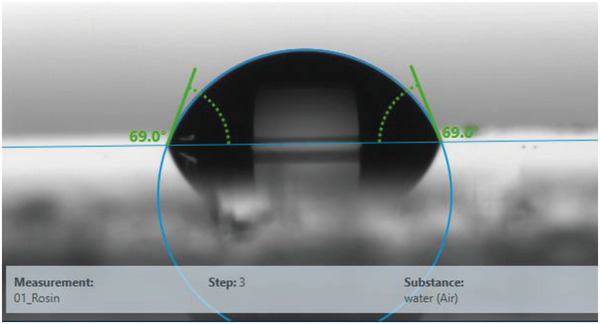
Contact angle of rosin resin. Left angle (water): 69.0°. Right angle (water): 69°. Surface energy 47.8 mN m^−1^ of which 40.4 mN m^−1^ as disperse component, and 7.4 mN m^−1^ as polar component.

**Figure 27:**
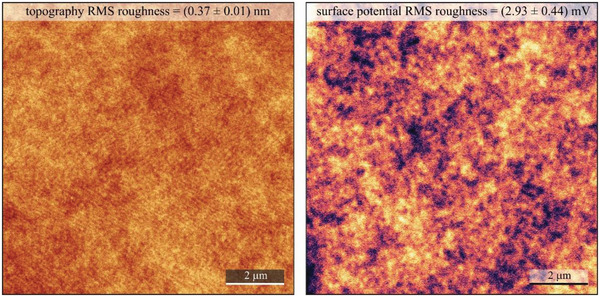
AFM and KPFM of rosin resin on gold‐coated glass. Left panel: 10 × 10 µm^2^ topography image of the investigated surface (*z* scale 5 nm). Right panel: corresponding surface potential map at the tip lift of 25 nm (*z* scale 20 mV). RMS roughness parameters are indicated for the corresponding images.

We performed surface investigation of rosin resin via contact angle measurement, AFM, and KPFM. The measured contact angle of around ≈69° with water droplet (see **Figure** **26**) and ≈38.5° with diiodomethane droplet (data not included) indicated a weakly hydrophilic surface for rosin resin, similar as the ones of black pine and shore pine resins. Total surface energy of rosin was 47.8 mN m^−1^, divided into a disperse component 40.4 mN m^−1^ and a polar component 7.4 mN m^−1^. In comparison to the other three investigated pine resin films, rosin samples were found to have the smallest surface roughness with almost atomically smooth surfaces (0.37 ± 0.01) nm, as shown in **Figure** **27**. However, spatial variations of the CPD, (2.93 ± 0.44) mV, were found to be comparable to most other resin films investigated here.

**Figure 28:**
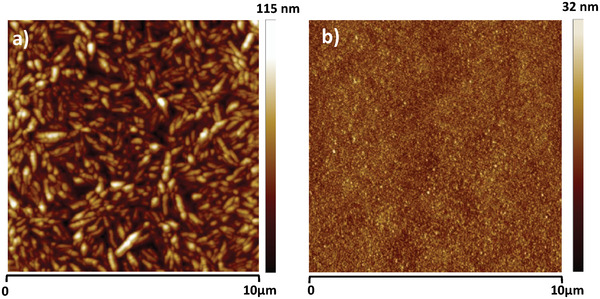
AFM measurements of a) pentacene on rosin dielectric, RMS ≈ 13 nm and b) fullerene (*C*_60_) grown on rosin dielectric, RMS ≈ 3.5 nm.

The topography of the semiconductors grown on rosin resin is presented in **Figure** **28**. In a similar fashion with the other analyzed resins in this study, pentacene grew in randomly oriented, large and elongated, dendritic grains of multimicron size and a surface roughness, RMS ≈13 nm, whereas *C*_60_ formed very small grains of several nanometers in size and a surface roughness, RMS ≈3.5 nm (see Figure 28).

#### 2.3.5 Dielectric Investigation

**Figure 29:**
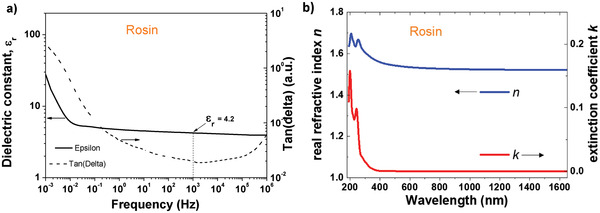
a) Impedance spectroscopy of rosin dielectric; b) complex refractive index *n*, *k* of rosin.

We performed dielectric measurements on a thin film of 417 nm thickness of rosin resin spin coated and dried on 1 mm wide aluminum electrode in a metal–insulator–metal configuration, with aluminum also as the top electrode material. We measured the dielectric spectroscopy for rosin between 1 MHz and 1 mHz and observed a very uniform capacitance over the measurement range, starting from the high frequency down to 10 mHz, followed by a slight increase in capacitance between 10 and 1 mHz. Likewise, the loss angle (tangent delta) shows no relaxation behavior over the entire measurement range (see **Figure** **29**). Both events are indicative of a very high purity dielectric film, with good dielectric performance. From the capacitance of the film at the frequency of 1 kHz, and given the thickness of the film of 417 nm, we calculated a dielectric constant of 4.2 for rosin, with a standard deviation of ± 0.2 for the 16 samples that were included in the study. The film of 417 nm thick rosin broke at 228 V, which corresponds to a breakdown field of 5.4 MV cm^−1^. Importantly, the entire set of 16 analyzed MIM structures consisted of films cast from the same solution and at identical rotation speed, and consequently the variation of thickness was consequently minimal. We did not pursue with this respect a typical study of breakdown field that takes into consideration the variation of the thickness of the dielectric.

The complex refractive index of rosin behaves similarly to the respective one of black pine resin, with its real part lying around 1.6, as shown in Figure 29b.

#### 2.3.6 Electrical Measurements

We fabricated field effect transistors on a commercially available rosin resin capped aluminum oxide (anodized to 10 V, with a thickness of ≈18 nm on the gate electrode) as the bilayer gate dielectric layer. We fabricated devices with both pentacene and *C*_60_ semiconductors. The devices were capped by source and drain electrodes made from aluminum in case of fullerene and gold in case of pentacene.

We fabricated the pentacene OFETs from a stock solution of 20 mg mL^−1^ rosin and the fullerene devices from a stock solution of 100 mg mL^−1^ rosin concentration in ethanol, respectively. The resin layer was dried at 80 °C for 1 h in ambient air prior to the semiconductor's deposition. The typical thickness of the organic resin dielectric was in the range of 70 nm, and the specific capacitance of the combo layer was situated in the range of ≈30 nF cm^−2^ for pentacene devices. For the fullerene devices, the specific capacitance of the inorganic–organic layer was ≈15 nF cm^−2^ given by a thickness of ≈18 nm of the aluminum oxide and ≈135 nm rosin combo dielectric. The rosin capping layer for the device with *C*_60_ was spin coated at a speed of 3500 rpm.

**Figure 30:**
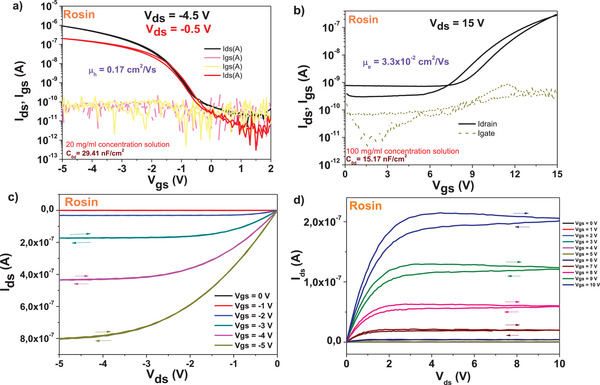
Transfer and output characteristics of rosin resin on AlOx dielectric with pentacene and *C*_60_ as organic semiconductors. a,b) are the transfer characteristics at two different drain voltages *V*_ds_ and c,d) are the output characteristics for gate voltages 0 ≤ *V*_gs_ ≤ 5 V of pentacene and 0 ≤ *V*_gs_ ≤ 10 V for *C*_60_ OFETs, respectively. The capacitance *C*_0d_ is noted in a) for pentacene and in b) for *C*_60_. Also indicated as insets are the hole and electron mobilities, *µ*_h_ and *µ*_e_ respectively, as well as the concentration of respective resins solutions in ethanol, from which the films were cast by spin coating.

Transfer and output characteristics of OFET devices with pentacene and *C*_60_ are presented in **Figure** **30**. In contrast with other resins investigated in this work, rosin offered an interface to pentacene suitable for a low level of OFF current in the range of 0.1 nA or lower (Figure 30a). The fullerene device at the same time displayed a much higher OFF level, in the range of 1 nA (Figure 30b). The two devices differed to one another also with respect to hysteresis. In line with other investigated resins in this work, pentacene‐based devices showed hysteresis‐free behavior in both transfer and output characteristics, whereas fullerene‐based devices displayed a significant hysteresis. The pentacene device recorded a very good field effect mobility of 0.17 cm^2^ V^−1^ s^−1^ and a very low subthreshold swing, in the range of 0.5 V dec^−1^. The normalized subthreshold swing of the device was 14.75 V nF cm^−2^ dec^−1^. The fullerene device was less performant, with significant hysteresis both in output and transfer characteristics (Figure 30b–d), one magnitude lower field effect mobility, i.e., 0.033 cm^2^ V^−1^ s^−1^ as well as significantly higher subthreshold swing, i.e., 2.1 V dec^−1^. and a normalized subthreshold swing of 31.8 V nF cm^−2^ dec^−1^.

**Figure 31:**
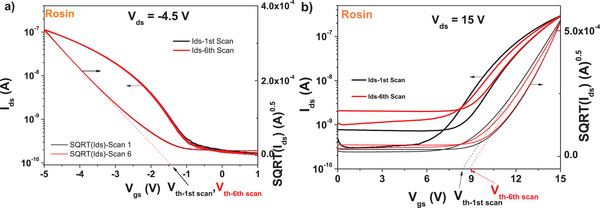
Rosin resin as dielectric with a) pentacene and b) *C*_60_ measured for stability under consecutive scanning. *V*_th_ stands for threshold voltage.

Rosin dielectric worked very well in the consecutive scanning test of the p‐type OFET device. As demonstrated in **Figure** **31**a), 6 consecutive scans of the device showed insignificant change on the transistor parameters, on all the ON and OFF levels as well as the threshold voltage. In the same time, in line with the lower performance obtained by the OFETs with fullerene channel showed in Figure 30b,d, also the consecutive scanning of rosin based OFET device proved inferior to its p‐type counterpart (Figure 31b). In the case of fullerene‐based device, an alteration of both ON and OFF levels of the device, as well as a shift of ≈600 mV of the threshold voltage occurred after only 6 consecutive measurements.

**Figure 32:**
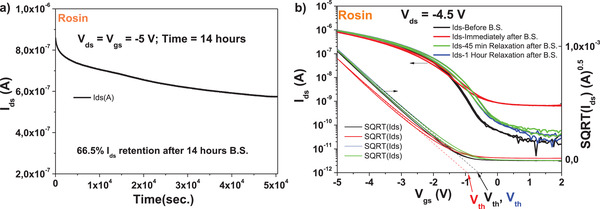
a) Bias stress and b) recovery after bias‐stress of the OFET characteristics with rosin dielectric and pentacene semiconductor.

We performed also bias stress measurements for rosin based OFETs with pentacene semiconductor and observed a ≈66.5% *I*_ds_ retention after stressing the device for 14 h at the maximum permitted voltages of drain and gate before breaking occurred, i.e., −5 V in case of the p‐type device presented in Figure 30. The rosin device did not fully recover after bias stress (see **Figure** **32**b, we measured the recovery with 5 min increment, but present only few curves to avoid burdening the figure), although it recovered within 15 mV voltage difference with respect to its original *V*_th_, and also within 50 pA difference of the OFF level after 1 h relaxation from the completion of the bias stress experiment. If we consider that both values described above of the *V*_th_ and OFF level are in fact negligible, the rosin device can be considered as having recovered after 1 h relaxation time.

### 2.4 Baltic Amber (*Pinus Succinifera*)

#### 2.4.1 Resin Type and Composition

**Figure 33:**
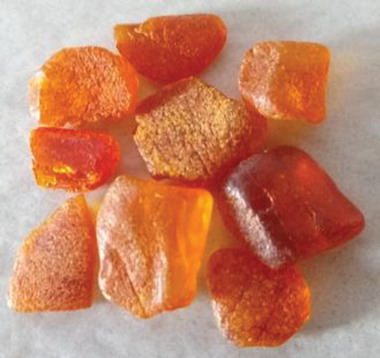
Photograph of Baltic amber nuggets collected from the Lithuanian shore of the Baltic sea.

Baltic amber nuggets available for research in our laboratory were collected from the Lithuanian shore of Baltic Sea in the year 2010. The image of the Baltic amber pellets is presented in **Figure** **33**.

Baltic amber is also called succinite, which indicates that it consists mainly of succinic acid‐related compounds. Several authors have assigned the source of Baltic amber to the genus *pinus succinifera*.^[^ ^111^, ^112^, ^113^, ^114^, ^115^ ^]^ However, according to Mosini and Samperi,^[^ ^116^ ^]^ Baltic amber may originate from “aged” *pinus halepensis* because *p. halepensis* resin gives products characteristic of Baltic amber (e.g., fenchyl alcohol and borneol) after an ageing process. Ambers contain both low‐molar‐mass compounds such as succinic acid and monoterpenes and larger molecules such as polymerized diterpenes.^[^ ^117^ ^]^ The ether soluble acid fraction of Baltic amber has been shown to contain large amounts of resin acids such as isopimaric acid (33%), dehydroabietic acid (21%), isopimaradienic acid (20%), abietic acid (16%), and abietadienic acid (10%).^[^ ^116^ ^]^ Baltic amber has also been shown to contain, e.g., retene.^[^ ^118^ ^]^ In the Baltic amber ethanol solution used in the present study, borneol (2.4%), pimaradienoic acids (1.9%), cholestadiene (1.5%), ethyl and diethyl succinate (1.3%), and diacetone alcohol (1.2%) dominated. Unidentified peaks accounted for 6.6% of the extract, and compounds not eluting on GC accounted for 82% of the extract. Of the unidentified GC eluting compounds, some may be succinates not present in the MS databases. The noneluting compounds is most probably oligomerized and polymerized material. In addition to GC analysis, we also performed high‐performance liquid chromatography (HPLC) and size exclusion chromatography for the Baltic amber resin, and the findings are presented in Tables 2 and 3 respectively. The chemical structure of the most prominent constituents of Baltic amber resin is presented in Figure 1.

#### 2.4.2 Structural Characterization

**Figure 34:**
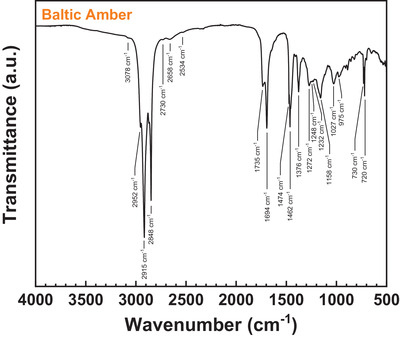
ATR‐FTIR spectrum of Baltic amber.

The ATR‐FTIR spectrum of the Baltic amber sample recorded from the solid material deposited from its solution in ethanol is shown in **Figure** **34**. Like the other resin materials investigated in this study, Baltic amber shows IR absorption features which are typical for carboxylic acids. These include the very broad carboxylic acid O–H stretching vibration band at 3600–2500 cm^−1^, the low intensity carboxylic acid overtone bands at 2658 and 2534 cm^−1^ and the C–O stretching band of the COOH group at 1694 cm^−1^. These bands can be assigned to the resin acid compounds^[^ ^75^, ^76^, ^77^ ^]^ present in the material, such as isopimaric acid and pimaradienoic acid as determined by GC–MS analysis. However, the carboxylic acid bands in the FTIR spectrum of Baltic amber show a lower relative intensity as compared to the resin acid‐dominated black pine, rosin, silver fir and Rocky Mountain fir resins, the latter two being part of our previous study.^[^ ^103^ ^]^ This coincides well with the results from GC–MS analysis that indicate a significant share of resin acids in the material, which however, do not comprise the dominating fraction of constituents. As opposed to the other investigated resins, amber shows a larger relative intensity of the methylene and methyl C–H stretching vibrations at 2952, 2915, and 2848 cm^−1^ and the corresponding C–H bending vibrations at 1474–1376 cm^−1^, indicating a large fraction of saturated aliphatic units in the resin. Furthermore, a clearly distinguishable absorption band at 1735 cm^−1^ can be observed which can be assigned to the C ═ O stretching vibration of esters. These bands can be attributed to succinic acid esters that may be present in the resin as molecules such as ethyl or isobornyl succinates, as suggested by the identification of succinates and isoborneol by GC–MS, which have been reported as components of ambers.^[^ ^118^, ^119^, ^120^ ^]^ The absorption bands in the region between 1272 and 1027 cm^−1^ can be attributed to O–H and C–O vibrations of carboxylic acid or ester functional groups and in‐plane C–H bending in cyclic hydrocarbons. The observed band pattern corresponds well to that reported by Beck et al.^[^ ^121^ ^]^ for different amber resins. The authors assigned the pronounced band near 1150 to the C–O vibration in saturated aliphatic esters^[^ ^121^ ^]^ which further corroborates the presence of these compounds in our Baltic amber sample. In the low wavenumber region, two sharp bands at 730 and 720 cm^−1^ are visible which we could unfortunately not clearly relate to compounds identified by GC–MS.

#### 2.4.3 Thermogravimetric Analysis

**Figure 35:**
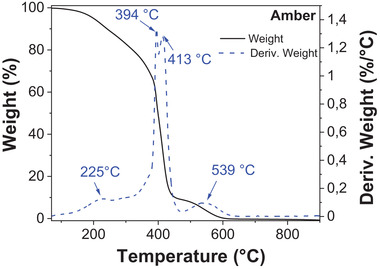
Thermogravimetric analysis of Baltic amber.

The thermal stability of the Baltic amber resin was analyzed by TGA using the same experimental heating setup than all the other resins. For this material (**Figure** **35**), a first weight loss was detected below 120 °C (0.27%) which corresponds to the adsorbed water from the resin. Then, a minor weight loss was centered at 225 °C, followed by the major and more pronounced weight loss between 300 and 450 °C, when the sample lost 88.8% of its weight, with two decomposition temperatures at 394 °C and 413 °C. From 450 to 650 °C another significant weight lost was detected (10.47%), followed by a final 0.46% weight loss until 900 °C, which left absolutely no residual weight, demonstrating a complete decomposition of the resin. Remarkably also, this resin showed the highest decomposition temperature, when compared with the other resins investigated in this study (see Figure 35 and **Table** **5**).

**Table 5:** Comparison of the resins with respect to their decomposition temperature, *T*_d_, and their weight loss divided over three ranges, from 120 to 900 °C. The weight loss below 120 °C was in the range of 0.9–1.1% for rosin, black pine and shore pine and 0.27% for Baltic amber

Resin *(stemming tree)*	*T*_d._ [°C]	Weight loss [%]	Weight loss [%]	Weight loss [%]	Residual material [%]
		120–450 °C	450–650 °C	650–900 °C	
**Black pine** *(pinus nigra)*	263	88.22	9.12	0.54	1.12
**Shore pine** (*pinus contorta*)	245	93.17	1.63	3.9	0.48
**Rosin** *(Sigma‐Aldrich; blend of pine resins)*	260	95.43	3.06	0.34	0.06
**Baltic amber** (*pinus succinifera*)	394	88.8	10.47	0.46	0

#### 2.4.4 Surface Investigation

**Figure 36:**
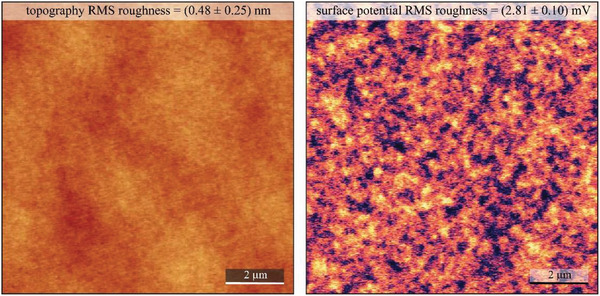
a) 10 × 10 µm^2^ topography image of the investigated Baltic amber resin surface (*z* scale 10 nm); b) corresponding surface potential map at the tip lift of 25 nm (*z* scale 20 mV). RMS roughness parameters are indicated for the corresponding images.

**Figure 37:**
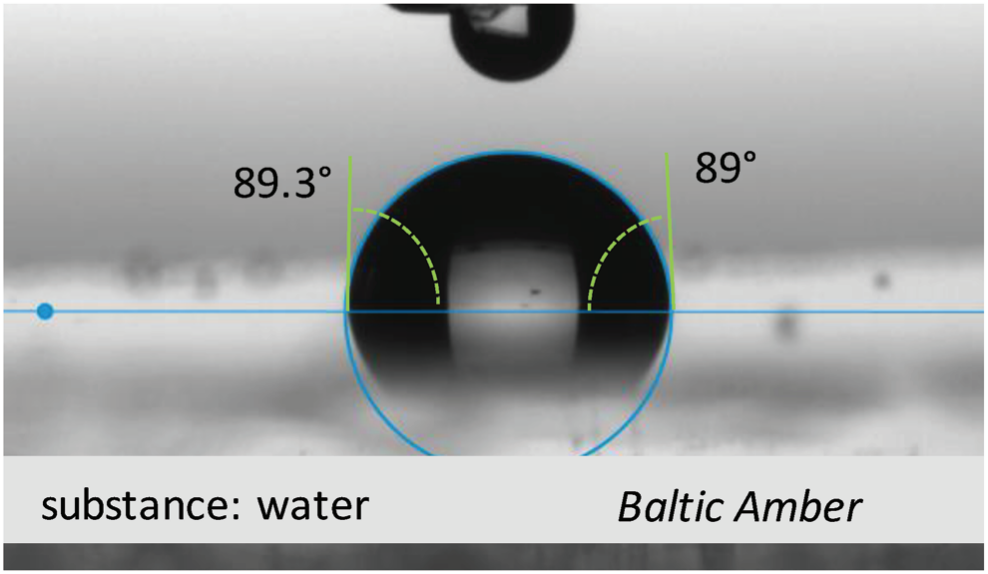
Contact angle of Baltic amber resin with water droplet as displayed in the figure. Left angle (water): 89.3°. Right angle (water): 89°. Surface energy 46 mN m^−1^ of which 0.5 mN m^−1^ as a polar component, and 45.5 mN m^−1^ as a disperse component. The measured contact angle with diiodo methane (not shown in the figure) was 26.8°.

We performed surface investigation of Baltic amber resin via AFM, KPFM (**Figure** **36**), and contact angle measurements (**Figure** **37**). Baltic amber films have shown similar high‐quality performance as most of the other investigated resin samples, and together with rosin, Baltic amber has shown the smallest surface potential variation and surface roughness. It is also worth and fair mentioning that in the case of Baltic amber, aging effects (as surface roughening and partial dewetting of the film) were observed after several months of ambient storing for the spin‐coated thin films on sputtered gold substrates.

The contact angle measurement shown in Figure 37 reveals slightly hydrophobic surface, with left and right measured contact angle of water droplet of 89.3° and 89° respectively. This makes amber the most hydrophobic surface among the four pine resins analyzed in this group of pinaceae tree resins.

**Figure 38:**
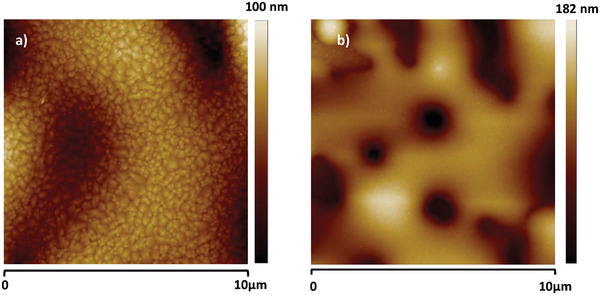
AFM scan of: a) pentacene semiconductor, RMS roughness ≈20 nm; and b) fullerene *C*_60_ on Baltic amber, RMS roughness ≈ 11 nm.

The topography of the semiconductors grown on Baltic amber fir resin is presented in **Figure** **38**. Pentacene grew in large elongated grains, of up to 350–400 nm in size and a typical RMS roughness of the top surface of the film in the range of ≈20 nm. Fullerene *C*_60_ deposited on Baltic amber grows in very small grains in size and formed a much smoother top surface, with an RMS roughness of ≈11 nm (Figure 38).

#### 2.4.5 Dielectric Spectroscopy

**Figure 39:**
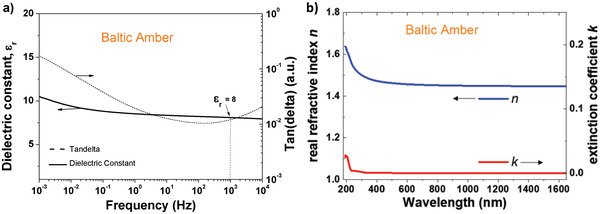
a) impedance measurement of Baltic amber; b) complex refractive index of Baltic amber.

We performed dielectric measurements on a film of 65 nm thick of Baltic amber resin spin coated and dried on 1 mm wide aluminum electrode in a metal–insulator–metal configuration, with aluminum as the top electrode material. We measured the dielectric spectroscopy between 10 kHz and 1 mHz and observed a very uniform capacitance all over the measurement range. Likewise, the loss angle (tangent delta) shows no relaxation behavior over the entire measurement range (see **Figure** **39**). Both events are indicative of a very high purity dielectric film, with good dielectric performance. From the measured capacitance and the thickness of the film given by profilometry investigation, we extracted a dielectric constant of 8 ± 0.3 for Baltic amber at 1 kHz for the investigated 16 MIM structures. The dielectric constant of 8 for the investigated amber thin films was really impressive, therefore we conducted a more careful study where we spin coated various thicknesses of the film and also carefully dried the respective films in vacuum to avoid any interference of the adsorbed moisture in the film over the capacitance measurement. However, we reached the same value of 8 as the dielectric constant for our thin film processed Baltic amber from ethanol solution shown in Figure 39.

We conducted also breakdown film measurements for amber in various film thicknesses. For the particular case of the ∼ 65 nm thick film, we obtained a value of ≈5.6 MV cm^−1^; however, this value was not correlated or confirmed yet from a wider study that considers also the thickness variation of the amber films. The isotropic complex refractive index, *n* and *k*, of Baltic amber is displayed in Figure 39b. It is very similar to the other resins but shows almost no absorption within the investigated UV spectral range.

#### 2.4.6 Electrical Measurements

**Figure 40:**
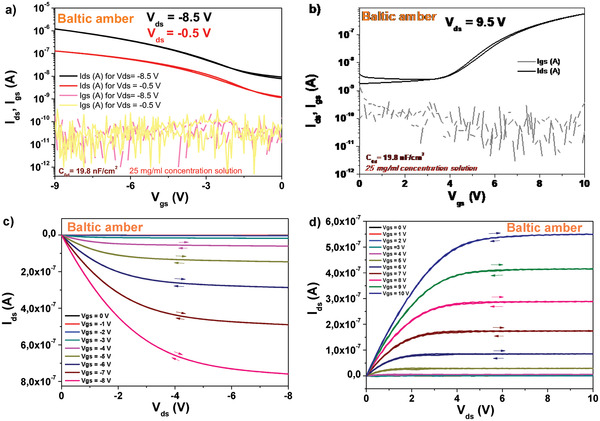
Transfer and output characteristics of Baltic amber resin on AlOx dielectric with pentacene and *C*_60_ as organic semiconductors. a,b) are the transfer characteristics at indicated drain voltages *V*_ds_; c,d) the output characteristics for gate voltages 0 ≤ *V*_gs_ ≤ −8 V for pentacene and 0 ≤ *V*_gs_ ≤ 10 V for *C*_60_ OFETs, respectively. The capacitance *C*_0d_ is noted in a) for pentacene and in b) for *C*_60_ as well as the concentration of respective resins solutions in ethanol. The hole (*µ*_h_) and electron (*µ*_e_) mobilities were 3.2 × 10^−2^ cm^2^ V^−1^ s^−1^ and 3.4 × 10^−2^ cm^2^ V^−1^ s^−1^ for pentacene and *C*_60_, respectively.

We fabricated field effect transistors on Baltic amber resin capped aluminum oxide gate electrode. We fabricated devices with both pentacene and *C*_60_ semiconductors. The devices were capped by source and drain electrodes made from aluminum in case of fullerene and gold in case of pentacene. The combo dielectric layer comprised electrochemically grown aluminum oxide inorganic layer (anodized to 10 V, having a thickness of ≈18 nm) capped by a thin layer of Baltic amber resin cast from a 20 mg mL^−1^ stock solution that was spin coated at a speed of 1500 rpm, then subsequently dried at 80 °C for 1 h in air on top of a hot plate. The specific capacitance *C*_0d_ of the bilayer was situated in the range of ≈19.8 nF cm^−2^. The device specific *C*_0d_ values are indicated in **Figure** **40**a,b. The typical transistor characteristics of Baltic amber resin dielectric showed hysteresis free behavior both in transfer and output characteristics for pentacene as well as for *C*_60_ semiconductors (see Figure 40a,d). The dielectric behavior was characterized by low leakage in the range of 10 to 100 pA all throughout the measurement range (i.e., 0 V to 10 V for fullerene semiconductor and 0 to −9 V for pentacene semiconductor respectively). The calculated semiconductor mobility was in the range of 0.03 cm^2^ V^−1^ s^−1^ for both type of semiconductors involved in the study. A characteristic of Baltic amber resin was the inducing of a relatively high OFF level of the organic transistor characteristics (in the range of 10^−8^ A for pentacene and 3 × 10^−8^ A for *C*_60_), possibly because of the tendency of the dielectric material to charge the two semiconductors in their OFF state. This finding is not surprising, and correlates well with the behavior of the other three pine resins with respect to the high level of the OFF level in the transfer characteristics of the OFETs.

**Figure 41:**
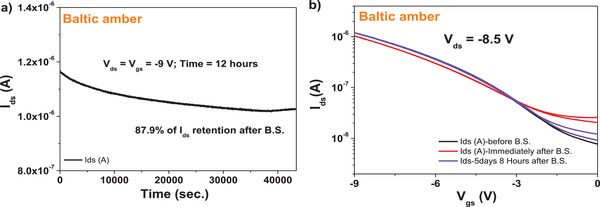
a) Bias stress and b) recovery after bias‐stress of Baltic amber OFET with pentacene semiconductor.

The subthreshold swing of the two semiconductors deposited on Baltic amber capping layer of AlOx dielectric was 3.5 V dec^−1^. for pentacene and 4 V dec^−1^. for fullerene. In the same time, the normalized subthreshold swing values were 69.5 V nF cm^−2^ dec^−1^. for pentacene and 79.2 V nF cm^−2^ dec^−1^. for *C*_60_. As it was the case with the calculated field effect mobility, also the normalized subthreshold swing was in the same value range for both pentacene and fullerene transistors. We performed bias stress measurement of the pentacene‐based OFET used for transfer and output characteristics and the respective results are displayed in **Figure** **41**.

We stressed the device at the maximum voltage used for transfer measurement (i.e., −9 V), while keeping both these drain and gate voltages constant for 12 h stress time. We measured the transfer characteristic at the beginning of the test, as well as immediately after releasing the electrical stress, and recorded a ≈88% retention of the *I*_ds_ current after releasing the bias stress. We continued to measure the recovery curve of the devices with 5 min increment, but for the simplicity and the avoidance of cluttering the graph, we show only the transfer curve where the full or nearly full recovery was measured. In the case of Baltic amber, 95.8% of the *I*_ds_ (ON level) was recovered in ≈3 h after completion of the bias stress, but the full recovery to the values measured before BS took 5 days and 8 h (see Figure 41b). Bias stress measurements with *C*_60_ provided more modest results (data not shown), with Amber nearly losing entirely the ON–OFF ratio of the transfer characteristics at the end of the testing period of 12 h. Again, this finding is not a surprise, and was observed for all the resins analyzed as part of this study or a previous one involving fir resins.^[^ ^103^ ^]^

**Figure 42:**
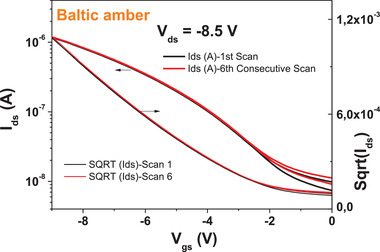
Stability after 6 consecutive scanning of the transfer characteristic of Baltic amber capping layer for Al_2_O_3_ with pentacene semiconductor.

We performed also the brief stability under consecutive scanning experiment for pine‐based OFETs with pentacene semiconductor (see **Figure** **42**), and observed that Baltic amber resin behaves very good with respect to this type of measurement stability, with a negligible shift of the threshold voltage after only 6 consecutive scans.

## 3 Discussion

**Figure 43:**
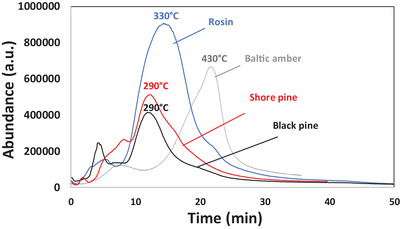
Thermograms, obtained by EGA‐MS, of the four investigated resins.

In this study we followed a curing protocol for the four analyzed resins that is successfully established in our laboratory, i.e., using a curing temperature of 80 °C for 1 h. We did not investigate the effect of curing at various other higher temperatures and for various amount of times as reported elsewhere.^[^ ^122^, ^123^ ^]^ Whether the resins films crosslink at higher temperature and if they change the surface morphology during crosslinking, will be determined in a future, more focused study that will be meant to reveal also the compositional changes in the films that occur during the drying process. Our immediate interest was to provide a robust dielectric layer for further exploitation in OFET devices, and the curing process selected clearly proved its value. The four pine resins analyzed in this work displayed many similarities among them, but also showed clear differences. The odd one out was Baltic amber, who presented marked differences in its appearance (stone like) and its processability compared to the other three. Baltic amber is a fossilized resin, of an extinct tree, *pinus succinifera* that grew in the Eocene and Oligocene periods just prior to the Ice Age, about 55 million years ago^[^ ^114^ ^]^ and is presently found in sedimentary rocks. Baltic amber showed in fact distinct dielectric properties, with much higher dielectric constant and also higher temperature of decomposition compared to the other 3 resins. In addition to the TGA analysis presented in respective chapters dealing with our analyzed pine resins, we also performed evolved gas‐mass spectrometry analysis (EGA‐MS, see **Figure** **43**). EGA‐MS analysis demonstrates that rosin, shore pine and black resin do not contain any polymerized fraction. The maximum temperature (at around 300 °C) of the peaks in the thermogram corresponds to desorption phenomena and not to pyrolysis processes. On the other hand, amber mainly contains polymeric fractions as we can argue from the high value of the maximum temperature (at around 430 °C) of the peak. These data agree with those obtained by solubilizing the resins in ethanol. Actually, amber showed a lower solubility than the other three resins. The results of EGA‐MS complement nicely the observations of thermogravimetric analysis, and show that Baltic amber contains indeed highly polymerized fractions. Interestingly, Baltic amber is the only resins not to leave any residue at the end of the TGA experiment that was run up to 900 °C. On the other hand, rosin that was industrially purified by distillation, left a very small residual material (0.06%, see Table 5) that is still comparable to the one of shore pine (0.48%). This is a very interesting observation considering that the TGA experiment was carried out with samples of each resin material extracted directly from their nuggets; consequently shore pine was not purified at all, not even solubilized and filtered, as was the case of the cast thin films. The comparative results of thermogravimetric analysis are presented in full in Table 5.

EGA‐MS analysis (Figure 43) demonstrates that rosin, shore pine and black pine resin do not contain any polymerized fraction. The maximum temperature of the peaks corresponds to desorption phenomena and not to pyrolysis processes. On the other hand, Baltic amber contains polymeric fractions as this fact can be interpreted from the high value of the maximum temperature of the peak.

**Figure 44:**
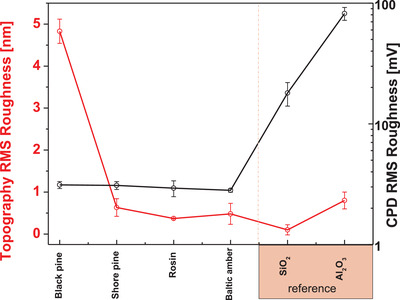
A comparison between topography RMS surface roughness (red, left scale bar) and CPD variations (black, right scale bar). The CPD RMS values are presented in a semi‐log, while topography RMS values are presented in a linear scale.

**Figure** **44** summarizes the RMS roughness values obtained by AFM and the RMS‐CPD fluctuations recorded via KPFM for all four pine resins; in addition, these values recorded for the cast films of pine resins are compared to the wet thermal SiO_2_ and anodized Al_2_O_3_ layers, that although being inorganic in nature, are still two of the most commonly used dielectrics in organic electronics studies. Identical procedures have been employed to obtain the RMS data for topography and CPD of the reference sample, as described for the pine resins in the Experimental Section. The two inorganic reference samples were found to have comparable or even smoother surfaces than the analyzed resin films, but their lateral variation of the surface potential was more than one order of magnitude larger than the one of all the four pine resins. These potential fluctuations could lead to formation of the interfacial dipoles, resulting in pronounced hysteresis between forward and reverse scans in the electrical transfer curves of the OFETs.^[^ ^124^ ^]^ A most likely reason for electrostatically more uniform surface of the pine resins is their less hydrophilic behavior than SiO_2_.^[^ ^125^, ^126^ ^]^

**Figure 45:**
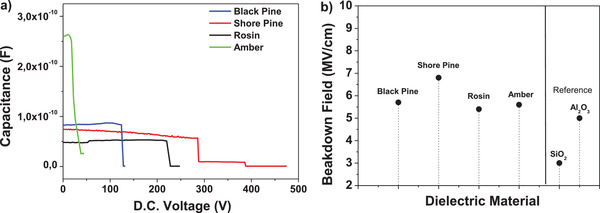
Breakdown field measured for the three pine resins. Black pine: thickness = 218 nm, breakdown voltage = 124 V, breakdown field 5.7 MV cm^−1^; Shore pine: thickness = 420 nm, breakdown voltage = 286 V, breakdown field 6.8 MV cm^−1^ for the first breakdown, 9.2 MV cm^−1^ for the final breakdown; Rosin: thickness: 417 nm, breakdown voltage: 228 V, breakdown field 5.4 MV cm^−1^; Baltic amber: thickness: 65 nm, breakdown voltage 38 V; breakdown field 5.6 MV cm^−1^.

A collection of the dielectric properties of the resins as well as the figures of merit of the fabricated OFETs containing the pine resins layers are presented in **Table** **6** below. Originating from different parts of the world (i.e., Idaho‐USA, Steiermark‐Austria, or Lithuanian shore of the Baltic sea), the three collected and the one purchased (rosin) pine resins were not only different in their composition revealed by Tables 1 and 2 and demonstrated by the FTIR measurements, but also behaved indeed differently in their overall performance, as Table 6 data shows. The only close similarity was the dielectric constant of rosin, black pine and shore pine, and in a way, the values of the breakdown field, which were remarkably high. With this respect, for all investigated pine resins, the dielectric strength values in excess of 5 MV cm^−1^ is remarkable (**Figure** **45**), when taking into account that the breakdown field of anodized Al_2_O_3_ is also no higher than 5 MV cm^−1^.^[^ ^127^ ^]^ To put that into context, for typical low‐k polymer dielectrics, the reported breakdown field is in the range of 1–2 MV cm^−1^, whereas for trimethylsilyl cellulose (TMSC), another low‐k bio‐based dielectric that was used in the fabrication of high performing OFETs and that can be regenerated to pure cellulose, a breakdown field of about 4.5 MV cm^−1^ was reported.^[^ ^128^ ^]^ In the same time, synthetic polymer resins like benzocyclobutene (BCB) have a breakdown field or dielectric strength no higher than 4.5 MV cm^−1^.^[^ ^129^, ^130^ ^]^ That means that the high purity of the pine resin dielectrics that is indicated by a low loss angle above 100 mHz and a corresponding flat capacitance translates also into a very high electric breakdown field. Only the shore pine film displayed higher levels of the loss angle at frequencies below 100 mHz, but this event was not translated into a relaxation of the respective coefficient (appearance of a dome shape in the characteristic). In fairness we have to point out though, that all the 16 MIM samples for each of the 4 resins evaluated for dielectric measurements (impedance spectroscopy and breakdown field) had similar dielectric thickness in each group, since they were processed from the same solution for each resin and deposited at the same rotation speed. It is well known that the breakdown voltage depends on the thickness of the film following the equation *V* = *At* ^2/3^, where *A* is a material constant and *t* is the thickness of the film. Therefore, as the thickness of the dielectric increases, the dielectric strength decreases. Therefore, the values reported in this work regarding the breakdown field may need to be considered as a particular example for the thickness of the dielectric films employed, and consequently need to be re‐visited in a more systematic study, as demonstrated elsewhere.^[^ ^131^ ^]^

**Table 6:** Dielectric and semiconductor properties for the fabricated OFETs with pentacene and *C*_60_

Resin name	Surface property			Dielectric property			Semiconductor performance in OFET with AlOx + resin						
	Roughness (nm)/Contact potential difference [mV]	Contact angle [deg.]	Surface energy [mN m^−1^] (disperse/polar)	Dielectric constant [a.u.]	Loss angle at 10^−3^ Hz [a.u.]	Breakdown field [MV cm^−1^]	OFET parameters				Bias stress (pentacene only)		
							Mobility [cm^2^ V^−1^ s^−1^]		*S*_sw_ [V dec^−1^]		Time [h]/Voltage [V]	*I*_ds_ retention [%]	Recovery time [min]
							Hole channel	Electron channel	Hole channel	Electron channel			
**Black Pine**	4.8/3.1	68	6.9	4	3	5.7	3.8 × 10^−2^	5.6 × 10^−2^	0.9	0.8	14/(−9)	55.8	135
			42.2										
**Shore Pine**	0.6/3	78	3.7	5.1	10	6.8 (9.2)	4.2 × 10^−2^	1.5 × 10^−2^	1	1.3	14/(−4)	95.6	45
			40.3										
**Rosin**	0.37/2.9	69	7.4	4.2	2.1	5.4	0.17	3.3 × 10^−2^	0.5	2.1	14/(−5)	66.5	60
			40.4										
**Baltic Amber**	0.5/2.8	89	0.5	8	0.12	5.6	3.2 × 10^−2^	3.4 × 10^−2^	3.5	4	12/(−9)	87.9	180
			45.5 0.5										

The FTIR spectra measured for the investigated pine resin materials could be correlated well with the general composition of the resins as determined by GC–MS analysis. The relative intensities of the characteristic vibrational bands of carboxylic acid groups of resin acids and the phenolic substructures of lignan compounds qualitatively correspond well to the fractions of the respective compounds in the resin materials as measured by GC–MS. ATR‐FTIR spectroscopy thus allows the straightforward classification of the resins into mainly resin acid‐based materials such as black pine, Baltic amber and rosin, and into lignan‐rich resins like the investigated shore pine resin. With the respect of FTIR analysis, it seems that black pine and rosin have many similarities, which helps understanding the true origin of rosin itself as the commercially available compound originating from “pine tree” species, per Sigma‐Aldrich claim. Characteristic IR bands can be related to specific dominant compounds; however, the identification of specific constituents is generally difficult due to the complex compositions of the investigated resins.

When comparing the topography RMS roughness values between the different pine resins of this study we found decreasing roughness when going from black pine to shore pine, to Baltic Amber and finally to rosin surface. The latter two resin surfaces have a roughness well below 1 nm which, and as expected, give rise to large sizes of the pentacene crystallites^[^ ^132^ ^]^ grown on such smooth surfaces. Interestingly, the 10‐fold increase in grain size of pentacene does not result in a larger hole mobility for shore pine OFETs as compared to black pine OFETs. Nevertheless, we observe a much larger mobility and a lower swing in the rosin OFET than in the shore pine OFET although they have a very similar pentacene grain size. Since in the pentacene OFETs, the normalized subthreshold swing, which is indicative of the interface trap density, is much smaller for the rosin resin surface as compared to the shore and black pine surfaces, might also explain the reason for the charge carrier mobility being largest in the rosin based OFETs. Interestingly, those interface traps in black and shore pine do not contribute to hysteresis occurrence in their *I*–*V* characteristics, so they most probably originate from deep states in the band gap. The electron mobility on the other hand is quite comparable for all the four investigated resin‐based bilayer dielectrics, which is not surprising given the similarity in the *C*_60_ morphology.

Notably, the shore pine film displayed a high level of loss angle at low frequencies, but this event was not translated into a relaxation of the respective coefficient (appearance of a dome shape in the characteristic), and also was not responsible for the occurrence of hysteresis in OFETs with pentacene.^[^ ^133^, ^134^ ^]^ However, as was reported before, it might be that the higher dielectric constant and thus larger polarizability of the shore pine resin interface to the semiconductor decreases the hole mobility.^[^ ^135^ ^]^ Nevertheless, the shore pine resin itself proved extreme stability, both in solubilized thin film as well as dry resin over a three‐year period, as demonstrated by the impedance spectroscopy study performed in Figure 19. The four materials when interfaced with pentacene, displayed also good performance with respect to bias stress and bias stress recovery after 12 or 14 h of stressing, with shore pine being outstanding compared with the other three with respect to these types of electrical measurements. In the same time, bias stress measurements with *C*_60_ proved disappointing, with almost complete disappearance of the transfer characteristics and an *I*_ds_ retention below 20% of the original value (data not shown).

Noteworthy, impressive is also the virtually hysteresis‐free electric performance of all investigated materials, especially when interfacing them with the p‐type semiconductor pentacene, a fact observed also for fir resins recently reported by us.^[^ ^103^ ^]^ With this respect, the analyzed pine resins perform very well compared to other classic dielectrics employed as capping layer for aluminum oxide: parylene‐C, divinyltetramethyldisiloxane‐bis(benzocyclo‐butene) (BCB), low density polyethylene, or adenine in combination with aluminum oxide.^[^ ^136^ ^]^

In the solubilization process, the resin layer surface is composed of free, unconnected molecules of the components of each particular resin with a large amount of free surface energy. This high surface energy enables the resin molecules to interact strongly with the semiconductor molecules deposited on the respective layer, i.e., pentacene and fullerene. We argue that this event might explain the good behavior of the dielectric resin layer in organic field‐effect transistor performance with respect to hysteresis occurrence between forward and reverse scans, a similar fact being also observed by us for the animal resin Shellac.^[^ ^9^, ^137^ ^]^ Nevertheless, it is fair to observe that all the pine resins are more performant with respect to the occurrence of hysteresis‐free behavior when they are interfaced to pentacene rather than *C*_60_. The cause of this preference is not yet clear, but it is worth mentioning that it represented a general trend for nearly all the resins investigated in our laboratory, more than 30 in total, which will be part of our future publications.

## 4 Conclusions

We performed in this study a thorough materials analysis of four resins stemming from pinaceae pine trees, via a plethora of compositional, electrical and surface characterization techniques, and subsequently employed them in the production of field effect transistors. The combined results presented here suggest that pine resins are promising candidates for the development of sustainable electronics devices, since they are coming directly from nature, are completely nontoxic (even possessing pharmaceutical properties), and do not need any purification step in order to reach “electronic grade”‐other than solubilization in a green solvent (ethanol) and a subsequent filtration. As it was demonstrated in case of shore pine in this work and the two fir resins analyzed in our previous publication (silver fir and Rocky mountain fir),^[^ ^103^ ^]^ pine resins are very stable to degradation both in solid form (nugget) and in their corresponding solubilized form in ethanol over many years of storage. We dully demonstrated here that pinaceae pine resins are also a class of high‐quality dielectric materials displaying robust dielectric characteristics and resistance to degradation in air and during measurement under prolonged electrical stress. Interestingly, the differences in film forming and dielectric performance between an industrially available, purified by distillation pine resin, i.e., rosin and the other 3 resins handpicked from living trees, i.e., black pine and shore pine, or found washed away on the shore of Baltic sea, i.e., amber, are minor, if any. We show throughout the manuscript that rosin is not at all more spectacular than the other 3 resins that were collected directly from mother nature, simply solubilized in ethanol and filtered through a hydrophilic filter paper. As we showed already in our previous publication analyzing two fir resins,^[^ ^103^ ^]^ and as we will show gradually with the entire group of 32 plant resins analyzed in our laboratories, the purification steps are not necessary for plant resins; they can be used as is, immediately after a simple filtration through a filter paper of their respective solutions in ethanol. Circumventing purification for component materials is a crucial step toward reaching the cost‐conscious sustainable development of electronics. In passing should be mentioned that the Baltic amber material analyzed in this study and utilized for the fabrication of organic field effect transistors, given its estimated age of about 55 million years in the petrified pellet,^[^ ^112^, ^113^, ^114^ ^]^ represents, arguably, the oldest material ever employed in the fabrication of organic electronic devices. Thus, all things considered, the four pinaceae pine resins investigated in this study represent viable alternatives for the fabrication of sustainable, “green” electronics.

## 5 Experimental Section

The resins employed in this work were collected from living trees (i.e., *shore pine* and *black pine*), or purchased from Aldrich directly (*rosin*) and solubilized in pure ethanol (99.9%) in 0.1 g mL^−1^ concentration by heating the solution while stirring vigorously for 30 min at 50 °C. *Baltic amber* was handpicked from the Lithuanian shore of the Baltic sea, was crushed in a mortar and solubilized in pure ethanol by stirring the solution overnight at 50 °C. The solution of Baltic amber had however a significantly lower concentration than the other three resins, given the stone like nature of the resin and its inherently limited solubility. All the solutions were filtered through a Chromafil, 0.2 µm pore size, hydrophilic filter paper and then diluted to the concentration employed for depositing thin films in this work, i.e., 20 or 25 mg mL^−1^ for the first three resins. The solubility of Baltic amber in pure ethanol was in the range of 20 mg mL^−1^, calculated from weighing the residual, undissolved material. In its case, no further dilution was necessary. All the resin thin films investigated in this work for dielectric investigations or transistor fabrications were spin coated with a rotation speed of at least 2500 rpm and subsequently dried on the top of a hot plate in air for 1 h at a temperature not exceeding 80 °C.

For gas chromatography, the resin samples were dissolved in 2 mL ethanol (usually containing around 200 mg per sample; nevertheless, the amber solution contained ≈40 mg of dry resin). 100 µL was withdrawn from each solution and was transferred to a tarred (with a microbalance) in a 1.5 mL glass vial. The solvent was evaporated using a stream of nitrogen, and the vials were kept in a vacuum oven (at 40 °C) for ≈30 min. After cooling to room temperature, the vials were weighed for determination of the weight of the dry extract. Subsequently, a volume of 1.0 or 1.5 mL of acetone was added, and the vials were kept for ≈1 min in an ultrasonic bath. A volume corresponding to around 0.6 mg of each dry resin was withdrawn and transferred to a 6 mL test tube and 2 mL of a solution containing 40 µg each of four internal standards (ISs) was added. The solvent was evaporated to dryness using a stream of nitrogen in a 40 °C water bath. Silylation reagents were then added: pyridine‐BSTFA‐TMCS 20:80:20 µL, and the tubes were kept in an oven at 70 °C for 30 min. The solutions were transferred using Pasteur pipettes to 1.5 mL GC vials with a glass insert. Circa 1 µL of each sample was injected to the GC–MS. Identifications were done by comparing the mass spectra with those in spectra databases: NIST12/Wiley 11th and our own database. The peak areas were integrated and the concentration of each compound was calculated by dividing the peak area with that of the IS (heneicosanoic acid for all compounds eluting before cholestadiene; and cholesterol or betulinol for cholestadiene and lignans). Then, the result was multiplied by the added amount of IS, and the results was divided by the amount of dry resin taken for the analysis.

AFM and KPFM (amplitude modulated‐AM) investigations were performed using a Horiba/AIST‐NT Omegascope AFM system. Nu‐nano SPARK probes were used (spring constant ≈42 N m^−1^, resonant frequency ≈350 kHz, tip radius below 30 nm). For AFM/KPFM experiments, the chromium‐gold back electrode of the resin films was grounded. AM‐KPFM measurements providing contact potential differences (CPD)^[^ ^138^ ^]^ were carried out in a two‐pass mode, with the probe lifted by 25 nm in the second pass. Root mean square (RMS) data is proved for both the topography roughness and the CPD fluctuations as an average with a standard deviation considering five arbitrarily chosen 10 × 10 µm^2^ areas of each resin sample. Topography and CPD images were processed with the aid of the open‐source software Gwyddion v2.56. For topography images zero‐order line filtering was applied and leveling of the base plane, whereas for CPD images only zero order line filtering was applied.

Thermogravimetric studies were performed with the aid of a TGA/PerkinElmer Q5000 instrument, using platinum pans and scanning from 70 to 900 °C, with heating rate of 10 °C min^−1^ under nitrogen atmosphere (25 mL min^−1^). All the four pine resin samples (in an amount ranging from 5 to 15 mg) were analyzed employing identical experimental heating setup and procedure. The amount of material for each resin was extracted from their respective pellet (nugget).

Thermal evolved gas analysis‐mass spectrometry (EGA‐MS) analyses were performed on an EGA/PY‐3030D microfurnace pyrolyzer (Frontier Laboratories, Japan) coupled to a 6890‐gas chromatograph and a 5973‐mass spectrometric detector (Agilent Technologies, USA). In each experiment, ≈100 µg of sample were directly weighted in the sample holder and inserted in the pyrolysis furnace. The temperature of the furnace was then raised from 50 to 700 °C at 10 °C min^−1^. The temperature of the pyrolysis interface was kept 100 °C above the furnace temperature, up to a maximum of 300 °C. Injection was performed in split mode at 280 °C with a 20:1 ratio. Desorption/pyrolysis products are directly sent to the MS detector through an UADTM‐2.5N deactivated stainless steel capillary tube (Frontier Laboratories, Japan) at 300 °C. Helium (1 mL min^–1^) was used as carrier gas. The mass spectrometer was operated in EI positive mode (70 eV, *m/z* range 50–800). The ion source and quadrupole analyzer were at 230 and 150 °C, respectively. Phosphitylation of samples was performed adapting the method described by elsewhere.^[^ ^139^ ^]^ All samples were finely grounded as described for EGA‐MS analyses before treatment. Samples were dried overnight in an oven set at 40 °C and then transferred in a desiccator until they reached room temperature. A mixture of pyridine and CDCl_3_ (1.6:1 v:v ratio) was prepared and dried over molecular sieves. Using this mixture, a 0.1 m solution of the relaxation reagent, chromium (III) acetylacetonate (5 mg mL^−1^), and of internal standard, cholesterol (40 mg mL^−1^), was prepared. All solutions were stored in the dark. About 40 mg of each sample were dissolved in 0.5 mL of solvent solution in a vial under stirring. Then, 0.1 µL of the internal standard and relaxation solution was added, and solution was stirred for ≈5 h to achieve solubilization of the sample. 0.1 mL of 2‐chloro‐4,4,5,5‐tetramethyl‐1,3,2‐dioxaphospholane (TMDP) was added and the clear solution was kept under vigorous magnetic stirring for 30 min. The resulting solution was transferred into an NMR tube.

^31^P‐NMR spectra were recorded on a JEOL YH spectrometer with a probe operating at 202.468 MHz at 25 °C in CDCl_3_. Chemical shifts were calibrated from the ^31^P NMR signal at 132.2 ppm arising from the reaction product between residual water and TMDP. Spectra were quantitative and proton broadband decoupling was applied during the acquisition time. Cholesterol was used as an internal standard. Spectra were acquired with 100 ppm spectral width, 32 000 data points, 11 s relaxation delay, and 256 scans. The spectra were analyzed using JEOL Delta software. For the quantitative ^31^P‐NMR spectroscopy, the derivatization of hydroxyl groups was completed with 2‐chloro‐4,4,5,5‐tetramethyl‐1,3,2‐dioxaphospholane (TMDP). This method offers the opportunity to rapidly identify aliphatic alcohols, unsubstituted or substituted phenols and carboxylic acid groups in all samples.

For complex impedance investigations, resin layers have been prepared by spin coating and subsequent drying at 80 °C on fused silica substrates. The complex refractive indices of a few hundreds of nanometer thick resin layers on fused silica substrates have been determined by variable angle spectroscopic ellipsometry. For this a J.A. Woollam M‐2000 DI ellipsometer and the vendor provided software CompleteEASE were used following the same procedure as published previously.^[^ ^103^ ^]^ No statistical analysis was performed for ellipsometry, sample size *n* = 1. Two ellipsometric measurements per sample were acquired and fitted jointly with a single transmission intensity measurement per sample for parameter decorrelation.

The dielectric strength (breakdown field) of the resins was measured in metal–insulator–metal (MIM) configuration, Each of the four analyzed resins was spin coated on top of a 80 nm thick, 1 mm wide plain aluminum electrode, dried for 1 h in ambient air at 80 °C, and the device terminated by the deposition of another layer of 80 nm thick, 1 mm wide aluminum electrode in cross configuration (i.e., perpendicular) to the bottom electrode. The MIM device was connected via two measurement pins to an impedance analyzer (Novocontrol GmbH) having available a DC booster instrument working to ±500 V DC voltage. A DC voltage scan was subsequently applied to the MIM device, starting from 0 V, with an increment of 2 V and 2.5 s. delay time at each particular applied voltage. The breakdown voltage was considered as the one for which a sudden loss of at least two orders of magnitude of the dielectric capacitance was observed on the measurement display. In case of the analyzed resins, the breakdown voltage could be clearly determined, since the resins did not break progressively, but sudden, as will be shown in a graph in the text of this article. The breakdown field was calculated by dividing the breakdown voltage to the value of the film thickness obtained via profilometry. We analyzed only 4 glass slides (in total 16 MIM samples) for each of the four resins for both dielectric and breakdown field measurements and observed a very uniform reproducibility of the results among the samples of each particular resin. All the films were cast from identical resin concentration solutions for each of the four resins, therefore the breakdown field experiment does not consider the variation of the results with the thicknesses of the dielectric films. Impedance spectroscopy was performed on an impedance analyzer (Novocontrol GmbH) in a frequency range between 10 kHz and 1 mHz.

Gas chromatography (GC) and high‐performance liquid chromatography (HPLC), high‐resolution mass spectrometry (MS) analyses have been performed for all samples, complemented by pyrolysis‐GC–MS analysis. The molar mass distribution of the four pine resins was obtained by size exclusion chromatography. For HPLC‐MS: ≈10 mg of each sample was mixed with 1 mL of acetonitrile and sonicated for 5 min, centrifuged and the soluble fraction analyzed using a Thermo Scientific Surveyor HPLC system coupled to a LTQ Orbitrap Velos mass spectrometer. The compounds were separated on a Thermo Scientific Accucore C18 column (150 mm × 3.0 mm, 2.6 um particle size) using a gradient with two phases: a mobile phase A containing 0.1% formic acid in water and a mobile phase B containing 0.1% formic acid in acetonitrile, at a flow rate of 0.5 mL min^−1^; the elution gradient starting conditions were 95% A and 5% B. After 2 min allowed for equilibration, the proportion of phase B was increased to 20% at 8 min, to 40% at 12 min, to 60% at 15 min, and finally to 95% at 19 min and held constant for another 4 min. UV detection was completed by a photodiode array detector and the mass spectra were recorded with an atmospheric pressure chemical ionization interface in FT mode with a resolution of 30 000. In the case of pyrolysis‐GC–MS: in order to achieve better performance in the analysis of the four resins, the pyrolysis has been performed in the presence of tetramethylammonium hydroxide (TMAH, Fluka).^[^ ^140^ ^]^ The experiments were performed with the aid of a CDS Pyroprobe 5250 pyrolyzer (CDS Analytical Inc.) coupled to a Trace GC Ultra (Thermo Electron Corp.) equipped with a capillary column Restek RTX35 (30 m × 0.32 mm × 0.25 µm), and a quadrupole mass spectrometer MD 800 (Fisons Instruments). 5 µL of saturated aqueous TMAH solution were added to ≈100 µg of sample and pyrolysis was performed at 550 °C for 10 s, with the pyrolizer interface set at 300 °C and the injector at 280 °C. The GC column temperature conditions were set as following: the initial temperature 50 °C, hold for 2 min, increase with a ramp of 20 °C min^−1^ to 300 °C, and hold this temperature for 10 min. Helium gas flow was set to 0.8 mL min^−1^, the split flow was 14 mL min^−1^. Mass spectra were recorded under electron impact ionization at 70 eV electron energy in the range from *m*/*z* 15–400. Identification of the compounds was done by comparison of the mass spectra with NIST 2011 electronic library and literature.^[^ ^141^ ^]^ For size exclusion chromatography, the samples were mixed with tetrahydrofuran (THF) and the insoluble part removed by filtration.

Attenuated total reflection Fourier‐transform infrared (ATR‐FTIR) spectra were measured on a Bruker Vertex 80 FTIR spectrometer equipped with a Bruker Platinum ATR unit having a liquid nitrogen cooled mercury cadmium telluride (MCT) detector. All the spectra were recorded with a resolution of 1 cm^−1^ and averaging over 200 scans. The solid resin material evaluated via ATR‐FTIR was obtained from ethanolic solutions of the resins by depositing the resin on a glass substrate via drop‐casting, drying the resulting film at 80 °C and removing by scrapping the resin material from the substrate.

The gate electrode of the fabricated OFETs contained a layer of aluminum oxide and a thin film resin as capping layer. The aluminum oxide was grown electrochemically following a reported method^[^ ^142^ ^]^ that was optimized over the years in our laboratory.^[^ ^46^, ^92^, ^143^ ^]^ The thickness of the aluminum gate electrodes that were subsequently anodized was 80 nm for all the OFET devices, and the anodization was carried out in a clean room environment of class 6 certified according to ISO 14 644, suitable for nanostructuring, novel electronic components and sensors development. Critically important, the aluminum wire used for evaporation to obtain the gate electrode had a purity of 99.999% (ChemPUR GmbH) and the respective gate electrode layer was evaporated at a fast rate of 4–5 nm s^−1^, which is advantageous for generating a very smooth aluminum layer, and allowing the fabrication of a very high‐quality aluminum oxide via anodization process. The anodization voltage of this study was set to 10 V, while maintaining in this process a steady current of 15 mA. After reaching the 10 V compliance, the sample was allowed to slowly continue to anodize until the final current reached ≈4.5 µA. The anodization procedure, as well as the geometries of the devices employed in the MIMs and OFETs investigations are identical to the ones reported in our previous publication dealing with silver fir and Rocky mountain fir resins.^[^ ^103^ ^]^

The semiconductor materials, pentacene and *C*_60_ were purchased from Sigma‐Aldrich, purified one time by train‐sublimation, and deposited in a Physical Vapor Deposition (PVD) System, using identical recipes of deposition (vacuum level, temperature ramp, deposition rate of 0.2–0.3 Å s^−1^, etc.) for pentacene for all the four resins and for *C*_60_ for all the four resins respectively, to account for a final thickness of 60 nm of each of the two semiconductors. Also, we employed an identical transistor geometry for all the OFETs fabricated in this study, with a 2 mm wide gate electrode and a channel dimension of 35 µm length and 2 mm width. Similar to our previous report,^[^ ^103^ ^]^ also in this study we did not attempt to optimize the semiconductor deposition for each particular resin in order to obtain record hole or electron mobilities,^[^ ^136^, ^144^ ^]^ but fabricated three batches of twenty‐four OFETs for each of the four resins employed in the study and for each of the two semiconductors. All the fabricated OFETs were measured on a probe station situated in a glove box under nitrogen atmosphere. The definition and the determination of the transistor parameters, i.e., the ON/OFF, the threshold voltage, the subthreshold swing (*S*_sw,_) the normalized subthreshold swing (*S*_sn_), and the field effect mobility were amply described in our recent publication,^[^ ^103^ ^]^ according to the work of Newman et al.^[^ ^145^ ^]^

## Conflict of Interest

The authors declare no conflict of interest.

## Data Availability

The data that support the findings of this study are available from the corresponding author upon reasonable request.

## References

[1] J. J. Green , J. H. Elisseeff , Nature 2016, 540, 386. 27974772 10.1038/nature21005 PMC8186828

[2] J. Edberg , R. Brooke , H. Granberg , I. Engquist , M. Berggren , Adv. Sustainable Syst. 2019, 3, 1900050.

[3] Y. S. Choi , Y.‐Y. Hsueh , J. Koo , Q. Yang , B. Hu , Z. Xie , R. Avila , G. Lee , Z. Ning , C. Liu , Y. Xu , Y. J. Lee , W. Zhao , J. Fang , Y. Deng , S. M. Lee , I. Stepien , Y. Yan , J. W. Song , C. Haney , Y. S. Oh , W. Liu , H.‐J. Yun , A. Banks , M. R. MacEwan , G. A. Ameer , W. Z. Ray , Y. Huang , T. Xie , C. K. Franz , et al., Nat Commun 2020, 11, 5990. 33239608 10.1038/s41467-020-19660-6 PMC7688647

[4] Y. Wang , M. Li , J.‐K. Chang , D. Aurelio , W. Li , B. J. Kim , J. H. Kim , M. Liscidini , J. A. Rogers , F. G. Omenetto , Nat Commun 2021, 12, 1651. 33712607 10.1038/s41467-021-21764-6 PMC7955034

[5] R. Avila , C. Li , Y. Xue , J. A. Rogers , Y. Huang , Proc. Natl. Acad. Sci. USA 2021, 118, e2020398118. 33836613 10.1073/pnas.2026405118 PMC7980470

[6] W. Wang , S. Wang , R. Rastak , Y. Ochiai , S. Niu , Y. Jiang , P. K. Arunachala , Y. Zheng , J. Xu , N. Matsuhisa , X. Yan , S.‐K. Kwon , M. Miyakawa , Z. Zhang , R. Ning , A. M. Foudeh , Y. Yun , C. Linder , J. B.‐H. Tok , Z. Bao , Nat. Elect. 2021, 4, 143.

[7] W. Wang , K. Ouaras , A. L. Rutz , X. Li , M. Gerigk , T. E. Naegele , G. G. Malliaras , Y. Y. S. Huang , Sci. Adv. 2020, 6, eaba0931. 32998891 10.1126/sciadv.aba0931 PMC7527227

[8] Z. Ma , D. Kong , L. Pan , Z. Bao , J. Semicond. 2020, 41, 041601.

[9] M. Baumgartner , M. E. Coppola , N. S. Sariciftci , E. D. Glowacki , S. Bauer , M. Irimia‐Vladu , in Green Materials for Electronics, (Eds.: M. Irimia‐Vladu , E. D. Glowacki , S. Bauer , N. S. Sariciftci ), Wiley‐VCH, Weinheim 2017, Vol. 101.

[10] L. Willner , B. Willner , Trends Biotechnol. 2001, 19, 222. 11356284 10.1016/s0167-7799(01)01634-1

[11] R. M. Owens , G. G. Malliaras , Mater. Today 2010, 35, 449.

[12] D.‐H. Kim , N. Lu , R. Ma , Y.‐S. Kim , R.‐H. Kim , S. Wang , J. Wu , S. M. Won , H. Tao , A. Islam , K. J. Yu , T. Kim , R. Chowdhury , M. Ying , L. Xu , M. Li , H.‐J. Chung , H. Keum , M. McCormick , P. Liu , Y.‐W. Zhang , F. G. Omenetto , Y. Huang , T. Coleman , J. A. Rogers , Science 2011, 333, 838. 21836009 10.1126/science.1206157

[13] D.‐L. Wen , D.‐H. Sun , P. Huang , W. Huang , M. Su , Y.a Wang , M.‐D. Han , B. Kim , J. Brugger , H. X. Zhang , X.‐S. Zhang , Microsyst. Nanoeng. 2021, 7, 35. 34567749 10.1038/s41378-021-00261-2 PMC8433308

[14] S. Chen , J. Qi , S. Fan , Z. Qiao , J. Chuan , Y. Chwee , T. Lim , Adv. Health. Mater. 2021, 10, 2100116. 10.1002/adhm.202100116 33960133

[15] Y. W. Kwon , Y. S. Jun , Y.‐G. Park , J. Jang , J.‐U. Park , Nano Res. 2021, 14, 3070.

[16] M. H. Zulfiqar , M. Ul Hassan , M. Zubair , M. Q. Mehmood , K. Riaz , IEEE Sens Lett 2021, 5, 5500604.

[17] E. O. Polat , Adv. Mater. Technol. 2021, 6, 2000853.

[18] J.‐H. Kim , I. Lee , T.‐S. Kim , N. Rolston , MRS Bull. , Stretchable and Ultraflexible Organic Electronics, xx 2017, Vol. 42, p. 115.

[19] S. E. Root , S. Savagatrup , A. D. Printz , D. Rodriquez , D. J. Lipomi , Chem. Rev. 2017, 117, 6467. 28343389 10.1021/acs.chemrev.7b00003

[20] S.‐M. Lee , J.‐H. Kim , J.‐H. Ahn , Mater. Today 2015, 18, 336.

[21] M. Held , A. Pichler , J. Chabeda , N. Lam , P. Hindenberg , C. Romero‐Nieto , G. Hernandez‐Sosa , Adv. Sustainable Syst. 2021, 6, 2100035.

[22] B.‐G. Cho , S. R. Joshi , S. Lee , S.‐K. Kim , Y.‐B. Park , G.‐H. Kim , Polymers 2021, 13, 615.33670700

[23] D. Ohayon , S. Inal , Adv. Mater. 2020, 32, 2001439. 10.1002/adma.202001439 32691880

[24] N. Mittal , A. Ojanguren , M. Niederberger , E. Lizundia , Adv. Sci. 2021, 8, 2004814. 10.1002/advs.202004814 PMC8224425 34194934

[25] L. M. Cavinato , E. Fresta , S. Ferrara , R. D. Costa , Adv. Energy Mater. 2021, 2100520.

[26] Z. Zhao , Y. Dai , S. X. Dou , J. Liang , Mater. Today 2021, 20, 100690.

[27] W. Kang , L. Zeng , S. Ling , C. Zhang , Adv. Energy Mater. 2021, 11, 2100020.

[28] T. A. Faraco , H. O. X. de Silva , H. S. da Barud , T. C. de Ribeiro , I. O. Maciel , W. G. Quirino , B. Fragneaud , M. Cremona , O. G. Pandoli , C. Legnani , ACS Appl. Electron. Mater. 2021, 2333, 10.1021/acsaelm.1c00217.

[29] R. Zhuang , R. J. Xie , Adv. Mater. 2021, 33, 2005925. 10.1002/adma.202005925 33786872

[30] S. Saha , S. Dawood , P. Butreddy , G. Pathiraja , H. Rathnayake , RSC Adv. 2021, 11, 16698. 35479177 10.1039/d1ra01513c PMC9032199

[31] S. K. Ghosh , J. Park , S. Na , M. P. Kim , H. Ko , Adv. Sci. 2021, 2005010. 10.1002/advs.202005010 PMC8261503 34258158

[32] N. Ebrahimi , C. Bi , D. J. Cappelleri , G. Ciuti , A. T. Conn , D. Faivre , N. Habibi , A. Hošovský , V. Iacovacci , I. S. M. Khalil , V. Magdanz , S. Misra , C. Pawashe , R. Rashidifar , P. E. D. Soto‐Rodriguez , Z. Fekete , A. Jafari , Adv. Funct. Mater. 2021, 31, 2005137.

[33] C. Xu , N. Kandel , X. Qiao , M.d. I. Khan , P. Pratakshya , N. E. Tolouei , B. Chen , A. A. Gorodetsky , ACS Appl. Mater. Interfaces 2021, 13, 20938. 33938723 10.1021/acsami.0c18929

[34] Z. Lin , Z. Meng , H. Miao , R. Wu , W. Qiu , N. Lin , X. Y. Liu , ACS Nano 2021, 15, 5649. 33660992 10.1021/acsnano.1c00820

[35] A. Joshi , V. Panwar , Mater. Today 2021, 10647, 10.1016/j.matpr.2021.01.384.

[36] M. Reali , A. Camus , G. Beaulieu , J. De Angelis , C. Pellerin , A. Pezzella , C. Santato , J. Phys. Chem. C 2021, 125, 3567.

[37] P. Guerrero , T. Garrido , I. Garcia‐Orue , E. Santos‐Vizcaino , M. Igartua , R. M. Hernandez , K. de la Caba , Polymers 2021, 13, 416. 33525478 10.3390/polym13030416 PMC7866128

[38] I. Cunha , J. Martins , D. Gaspar , P. G. Bahubalindruni , E. Fortunato , R. Martins , L. Pereira , Adv. Electron. Mater. 2021, 7, 2001166.

[39] J. Wünsche , Y. Deng , P. Kumar , E. Di Mauro , E. Josberger , J. Sayago , A. Pezzella , F. Soavi , F. Cicoira , M. Rolandi , C. Santato , Chem. Mater. 2015, 27, 436.

[40] M. Irimia‐Vladu , Chem. Soc. Rev. 2014, 43, 588. 24121237 10.1039/c3cs60235d

[41] B. Stadlober , M. Zirkl , M. Irimia‐Vladu , Chem. Soc. Rev. 2019, 48, 1787. 30776029 10.1039/c8cs00928g

[42] M. P. Cenci , T. Scarazzato , D. D. Munchen , P. C. Dartora , H. M. Veit , A. Moura Bernardes , P. R. Dias , Adv. Mater. Technol. 2021, 7, 2001263.

[43] F. Hartmann , M. Baumgartner , M. Kaltenbrunner , „B. Sustainable , Adv. Mater. 2021, 33, 2004413. 10.1002/adma.202004413 PMC11468029 33336520

[44] M. B. Gawande , V. D. B. Bonifácio , R. Luque , P. S. Branco , R. S. Varma , Chem. Soc. Rev. 2013, 42, 5522. 23529409 10.1039/c3cs60025d

[45] A. Petritz , A. Wolfberger , A. Fian , T. Griesser , M. Irimia‐Vladu , B. Stadlober , Adv. Mater. 2015, 27, 7645 . 25898801 10.1002/adma.201404627

[46] M. Irimia‐Vladu , P. A. Troshin , M. Reisinger , G. Schwabegger , M. Ullah , R. Schwödiauer , A. Mumyatov , M. Bodea , J. W. Fergus , V. F. Razumov , H. Sitter , S. Bauer , N. S. Sariciftci , Org. Electron. 2010, 11, 1974.

[47] N. R. Misra , S. Kumar , A. Jain , International Conference on Computing, Communication, and Intelligent Systems, ICCCIS, xx 2021, pp. 1032–1036, ISBN: 978‐1‐7281‐8529‐3/21.

[48] M. Irimia‐Vladu , E. D. Glowacki , P. A. Troshin , D. K. Susarova , O. Krystal , G. Schwabegger , M. Ullah , Y. Kanbur , M. A. Bodea , V. F. Razumov , H. Sitter , S. Bauer , N. S. Sariciftci , Adv. Mater. 2012, 24, 375 . 22109816 10.1002/adma.201102619

[49] M. Irimia‐Vladu , Y. Kanbur , F.a Camaioni , M. E. Coppola , C. Yumusak , C. V. Irimia , A. Vlad , A. Operamolla , G. M. Farinola , G. P. Suranna , N. González‐Benitez , M. C. Molina , L. F. Bautista , H. Langhals , B. Stadlober , E. D. Głowacki , N. S. Sariciftci , Chem. Mater. 2019, 31, 6315. 32565617 10.1021/acs.chemmater.9b01405 PMC7297463

[50] H. J. Jina , S. H. Leeb , T. H. Kim , J. Park , H. S. Song , T. H. Park , S. Hong , Bios 2012, 35, 335.

[51] S. H. Lee , M. Lee , H. Y. , Y. Cho , S. Hong , T. H. Park , Bios 2020, 154, 112071. 10.1016/j.bios.2020.112071 32056965

[52] M. Gamella , N. Guz , J. M. Pingarrón , R. Aslebagh , C. C. Darie , E. Katz , Chem. Commun. 2015, 51, 7618. 10.1039/c5cc01498k 25846235

[53] J. R. Sheats , J. Mater. Res. 2004, 19, 1974.

[54] T. G. Gutowski , M. S. Branham , J. B. Dahmus , A. J. Jones , A. Thiriez , D. P. Sekulic , Environ. Sci. Technol. 2009, 43, 1584. 19350939 10.1021/es8016655

[55] E. Williams , Environ. Sci. Technol. 2004, 38, 6166. 15573621 10.1021/es035152j

[56] H. Sirringhaus , Adv. Mater. 2014, 26, 1319. 24443057 10.1002/adma.201304346 PMC4515091

[57] H. Klauk , Adv. Electron. Mater. 2018, 4, 1700474.

[58] B. Stadlober , M. Zirkl , M. Irimia‐Vladu , Chem. Soc. Rev. 2019, 48, 1787. 30776029 10.1039/c8cs00928g

[59] M. White , M. Kaltenbrunner , E. Glowacki , K. Gutnichenko , G. Kettlgruber , I. Graz , S. Aazou , C. Ulbricht , D. A. M. Egbe , M. C. Miron , Z. Major , M. C. Scharber , T. Sekitani , T. Someya , Nat Photonics 2013, 7, 811.

[60] P. Meredith , C. Bettinger , M. Irimia‐Vladu , A. Mostert , P. Schwenn , Rep Prog Phys 2013, 76, 034501. 23411598 10.1088/0034-4885/76/3/034501

[61] D. K. Khatu , N. P. Maria Joseph , R. G. Khandelwal , A. N. Rao , S.‐J. Kim , Mater. Today 2021, 20, 100679.

[62] S. Appusamy , S. Krishnan , M. Gopikrishna , S. Raman , J. Electron. Mater. 2021, 50, 1893.

[63] S. Xu , A. Jayaraman , J. A. Rogers , Nature 2019, 571, 319. 31316200 10.1038/d41586-019-02143-0

[64] H. U. Chung , B. H. Kim , J. Y. Lee , J. Lee , Z. Xie , E. M. Ibler , K. H. Lee , A. Banks , J. Y. Jeong , J. Kim , C. Ogle , D. Grande , Y. Yu , H. Jang , P. Assem , D. Ryu , J. W. Kwak , M. Namkoong , J. B. Park , Y. Lee , D. H. Kim , A. Ryu , J. Jeong , K. You , B. Ji , Z. Liu , Q. Huo , X. Feng , Y. Deng , Y. Xu , et al., Science 2019, 363, 6430. 10.1126/science.aau0780 PMC6510306 30819934

[65] A. A. Kumar , J. W. Hennek , B. S. Smith , S. Kumar , P. Beattie , S. Jain , J. P. Rolland , T. P. Stossel , C. Chunda‐Liyoka , G. M. Whitesides , Angew. Chem., Int. Ed. 2015, 54, 5836. 10.1002/anie.201411741 25914299

[66] L. M. Dumitru , M. Irimia‐Vladu , N. S. Sariciftci , Comprehens. Anal. Chem. 2016, 74, 247.

[67] G. M. Whitesides , The Economist—The World in 2012, 2011, p. 154.

[68] L. Wang , D. Chen , K. Jiangd , G. Shen , Chem. Soc. Rev. 2017,46, 6764. 28875205 10.1039/c7cs00278e

[69] P. Wang , M. Hu , H. Wang , Z. Chen , Y. Feng , J. Wang , W. Ling , Y. Huang , Adv. Sci. 2020, 7, 2001116. 10.1002/advs.202001116 PMC7578875 33101851

[70] J. H. Langenheim , Plant Resins: Chemistry, Evolution, Ecology and Ethnobotany, Timber Press Inc. , xx 2003.

[71] S. S. Negi , Forests for Socio‐Economic and Rural Development in India, MD Publications, New Delhi, 1996.

[72] Ç. Kizilarslan , E. Sevgi , Indian J. Trad. Med. 2013, 12, 209.

[73] İ. Gülçin , M. E. Büyükokuroǧlu , M. Oktay , Ö. I. Küfrevioǧlu , J. Ethnopharmacol. 2003, 86, 51. 12686441 10.1016/s0378-8741(03)00036-9

[74] S. Arı , M. Kargioğlu , M. Temel , M. Konuk , J Ethnobiol Ethnomed 2014, 10, 29. 24673846 10.1186/1746-4269-10-29 PMC3974423

[75] E. P. Favvas , E. P. Kouvelos , S. K. Papageorgiou , C. G. Tsanaktsidis , A. C. Mitropoulos , Appl. Phys. A 2015, 119, 735.

[76] W. Nong , X. Chen , J. Liang , L. Wang , Z. Tong , K. Huang , R. Wu , Q. Xie , Y. Jia , K. Li , Adv. Mater. 2014, 887–888, 551.

[77] V. Beltran , N. Salvado , S. Buti , T. Pradell , Anal. Bioanal. Chem. 2016, 408, 4073. 27052772 10.1007/s00216-016-9496-x

[78] J. Jakobsen , Spectrochim. Acta 1965, 21, 433.

[79] H. L. Hergert , J. Org. Chem. 1960, 25, 405.

[80] P. Bock , N. Gierlinger , J. Raman Spectrosc. 2019, 50, 778. 31263319 10.1002/jrs.5588 PMC6602882

[81] P. Jia , Y. Ma , G. Feng , L. Hu , Y. Zhou , J. Cleaner Prod. 2019, 227, 662.

[82] P. Jia , Y. Ma , G. Feng , L. Hu , Y. Zhou , J. Cleaner Prod. 2019, 227, 662.

[83] C. Tsanaktsidis , A. Stimoniaris , S. Bousios , G. Tzilantonis , A. Scaltsoyiannes , M. Taktsira , A. Scaltsoyiannes , J. Environ. Protect. 2016, 7, 583.

[84] P. Liu , X. Liu , T. Saburi , S. Kubota , P. Huang , Y. Wada , ACS Omega 2020, 5, 29102. 33225141 10.1021/acsomega.0c03736 PMC7675569

[85] M. Carrier , A. Loppinet‐Serani , D. Denux , J.‐M. Lasnier , F. Ham‐Pichavant , F. Cansell , C. Aymonier , Biomass Bioenergy 2011, 35, 298.

[86] E. Biagini , F. Barontini , L. Tognotti , Ind. Eng. Chem. Res. 2006, 45, 4486.

[87] C. G. Tsanaktsidis , E. P. Favvas , A. A. Scaltsoyiannes , S. G. Christidis , E. X. Katsidi , A. V. Scaltsoyiannes , Fuel Process. Technol. 2013, 114, 135.

[88] Impedance Spectroscopy: Theory, Experiment, and Applications, 2nd ed. (Eds.: E. Barsoukov , J. R. Macdonald ), John Wiley and Sons, xx 2005, 10.1002/0471716243.

[89] A. H. Alami , K. Aokal , D. Zhang , A. Taieb , M. Faraj , A. Alhammadi , J. M. Ashraf , B. Soudan , J. El Hajjar , Int. J. Energy Res. 43, 5824.

[90] M. Irimia‐Vladu , J. W. Fergus , Synth. Met. 2006, 156, 1396.

[91] M. Irimia‐Vladu , N. Marjanovic , M. Bodea , G. Hernandez‐Sosa , A. Montaigne Ramil , R. Schwödiauer , S. Bauer , N. S. Sariciftci , F. Nüesch , Org. Electron. 2009, 10, 408.

[92] C. Yumusak , N. S. Sariciftci , M. Irimia‐Vladu , Mater. Chem. Front. 2020, 4, 3678.

[93] G. Moore , B. Kershner , T. Craig , D. Mathews , G. Nelson , R. Spellenberg , J. W. Thieret , T. Purinton , A. Block , National Wildlife Federation Field Guide to Trees of North America , Sterling, New York 2008, ISBN 978‐1‐4027‐3875‐3.

[94] OECD , in Safety Assessment of Transgenic Organisms, OECD Consensus Documents, OECD Publishing, Paris, 2010, Vol. 3, 10.1787/9789264095434-9-en.

[95] R. Kral , in Flora of North America Editorial Committee (ed.). Flora of North America North of Mexico (FNA). 2. New York and Oxford. Retrieved 12 September 2010 – via eFloras.org, Missouri Botanical Garden, St. Louis, MO & Harvard University Herbaria, Cambridge, MA, 1993.

[96] S. Zhao , N. Erbilgin , Front Plant Sci 2019, 10, 1459. 31850006 10.3389/fpls.2019.01459 PMC6888816

[97] S. J. Martinson , A. A. Fernádez Ajó , A. S. Martínez , F. E. Krivak‐Tetley , J. M. Villacide , M. P. Ayres , J. C. Corley , Bull. Entomological Res. 2019, 109, 141. 10.1017/S0007485318000184 29665874

[98] B. D. Compton , Ph.D. Dissertation, University of British Columbia, xx 1993.

[99] S. Bisoyi , U. Zschieschang , M. J. Kang , K. Takimiya , H. Klauk , S. P. Tiwari , Org. Electron. 2014, 15, 3173.

[100] S. Park , E. N. Cho , I. Yun , Microelectron Reliab 2012, 52, 2215.

[101] U. Zschieschang , R. T. Weitz , K. Kern , H. Klauk , Appl. Phys. A: Mater. Sci. Process. 2009, 95, 139.

[102] J. Kim , J. Jang , K. K. , H. Kim , S. H. Kim , C. E. Park , Adv. Mater. 2014, 26, 7241. 25263950 10.1002/adma.201402363

[103] J. Ivić , A. Petritz , C. V. Irimia , B. Kahraman , Y. Kanbur , M. Bednorz , C. Yumusak , M. A. Aslam , A. Matković , K. Saller , C. Schwarzinger , W. Schühly , A. I. Smeds , Y. Salinas , M. Schiek , F. Mayr , C. Xu , C. Teichert , M. Osiac , N. S. Sariciftci , B. Stadlober , M. Irimia‐Vladu , Adv Sustain Syst 2022, 6, 2200234.

[104] B. Holmbom , in: Biorefining of Forest Resources (Ed.: R. Alén ), Paper Engineers’ Association/Paperi ja Puu Oy, Porvoo, Finland 2011, pp. 178–224.

[105] Estimate: Harima Chemicals Group, Inc. , https://www.harima.co.jp/en/pine_chemicals/rosin3.html

[106] Z. Song , Forest Chem. Rev. 1999, 109, 7.

[107] M. M. Joye Jr. , R. W. Lawrence , J. Chem. Eng. Data 1967, 12, 279.

[108] 2008 International Yearbook, Forest Chem. Rev. (Ed.: J. M. Turner ), Kriedt Enterprises, New Orleans, LA, USA 2010, p. 7.

[109] S. Palkin , W. C. Smith , Oil Soap 1938, 120, 138.

[110] K. Fiebach , D. Grimm , Ullmann's Encycl. Ind. Chem. 2012, 31, 485.

[111] V. Mosini , M. L. Forcellese , R. Nicoletti , Phytochemistry 1980, 19, 679.

[112] K. Schubert , Neue Untersuchungen über Bau und Leben der Bernsteinkiefern Pinus succifinifera (CONW.) emend. Beihefte zum Geologischen Jahrbuch, Heft 45, xx, Hannover 1961.

[113] C. Beck , E. Wilbur , S. Meret , D. Kossove , K. Kermani , Archaeometry 1965, 8, 96.

[114] L. Gough , J. Mills , Nature 1972, 239, 527.

[115] R. C. A. Rottlander , Archaeometry 1970, 12, 35.

[116] V. Mosini , R. Samperi , Phytochemistry 1985, 24, 859.

[117] N. Vávra , Ann. Naturhist. Mus. Wien. 111A, 445.

[118] J. S. Mills , R. White , L. J. Gough , Chem. Geol. 1984, 47, 15.

[119] F. Czechowski , B. R. T. Simoneit , M. Sachanbinski , J. Chojcan , S. Wolowiec , Appl. Geochem. 1996, 11, 811.

[120] J. Poulin , K. Helwig , Anal. Chem. 2014, 86, 7428. 24945921 10.1021/ac501073k

[121] C. Beck , E. Wilbur , S. Meret , D. Kossove , K. Kermani , Archaeometry 1965, 8, 96.

[122] J. Li , P. Guo , X. Kong , Y. Wang , Y. Yang , F. Liu , B. Du , IEEE Trans. Dielectr. Electr. Insul. 2023, 30, 20.

[123] J. Li , P. Guo , X. Kong , Y. Wang , Y. Yang , F. Liu , B. Du , IEEE Trans. Diel. Electr. Insul. 2022, 29, 2072.

[124] C. Yumusak , F. Mayr , D. Wielend , B. Kahraman , Y. Kanbur , H. Langhals , M. Irimia‐Vladu , Isr. J. Chem. 2022, 202100126.

[125] R. R. Thomas , F. B. Kaufman , J. T. Kirleis , R. A. Belsky , J. Electrochem. Soc. 1996, 143, 643.

[126] B. Vasić , C. Czibula , M. Kratzer , B. R. A. Neves , A. Matković , C. Teichert , Nanotechnology 2021, 32, 265701. 10.1088/1361-6528/abeffe 33735842

[127] B. Wang , W. Huang , L. Chi , M. Al‐Hashimi , T. J. Marks , A. Facchetti , Chem. Soc. Rev. 2018, 118, 5690. 10.1021/acs.chemrev.8b00045 29785854

[128] A. Petritz , A. Wolfberger , A. Fian , T. Griesser , M. Irimia‐Vladu , B. Stadlober , Adv. Mater. 2015, 27, 7645. 25898801 10.1002/adma.201404627

[129] A. Modafe , N. Ghalichechian , B. Kleber , R. Ghodssi , IEEE Trans. Device Mater. Reliab. 2004, 4, 495.

[130] M. Paeck , M. Woehrmann , M. Teopper , K. D. Lang , in 2019 IEEE 69th Electronic Components and Technology Conf. (ECTC), IEEE, Las Vegas, NV, USA 2019, 10.1109/ECTC.2019.00285.

[131] L. Zha , C. L. Liu , Nanomaterials 2020, 10, 2473.33321774

[132] S. Steudel , S. De Vusser , S. De Jonge , D. Janssen , S. Verlaak , J. Genoe , P. Heremans , Appl. Phys. Lett. 2004, 85, 4400.

[133] M. Egginger , M. Irimia‐Vladu , R. Schwödiauer , A. Tanda , I. Frischauf , S. Bauer , N. S. Sariciftci , Adv. Mater. 20, 1018.

[134] M. Egginger , M. Irimia‐Vladu , R. Schwödiauer , A. Tanda , I. Frischauf , S. Bauer , N. S. Sariciftci , Adv. Mater. 20, 1018.

[135] H. Sirringhaus , M. Bird , T. Richards , N. Zhao , Adv. Mater. 2010, 22, 3893. 20954269 10.1002/adma.200902857

[136] G. Schwabegger , M. Ullah , M. Irimia‐Vladu , M. Reisinger , Y. Kanbur , R. Ahmed , P. Stadler , S. Bauer , N. S. Sariciftci , H. Sitter , Synth. Met. 2011, 161, 2058. 22049252 10.1016/j.synthmet.2011.06.042 PMC3197884

[137] M. Irimia‐Vladu , E. D. Głowacki , G. Schwabegger , L. Leonat , H. Z. Akpinar , H. Sitter , S. Bauer , N. S. Sariciftci , Green Chem. 2013, 15, 1473.

[138] Y. Udum , P. Denk , G. A. Workneh , D. H. Apaydin , A. Nevosad , C. Teichert , M. S. White , N. S. Sariciftci , M. C. Scharber , Org. Electron. 2014, 15, 997. 24817837 10.1016/j.orgel.2014.02.009 PMC4010259

[139] D. S. Argyropoulos , N. Pajer , C. Crestini , J. Visual. Exp. 2021, 174, e62696. 10.3791/62696 34398158

[140] C. Schwarzinger , J. Anal. Appl. Pyrolysis 2003, 68–69, 137.

[141] I. Pastorova , K. J. van der Berg , J. J. Boon , J. W. Verhoeven , J. Anal. Appl. Pyrolysis 1997, 43, 41.

[142] L. A. Majewski , M. Grell , S. D. Ogier , J. Veres , Org. Electron. 2003, 4, 27.

[143] A. I. Mardare , M. Kaltenbrunner , N. S. Sariciftci , S. Bauer , A. W. Hassel , Phys. Status Solidi A 2012, 209, 813.

[144] Y. Kanbur , M. Irimia‐Vladu , E. D. Glowacki , M. Baumgartner , G. Schwabegger , L. N. Leonat , M. Ullah , H. Sitter , R. Schwödiauer , Z. Kücükyavuz , S. Bauer , N. S. Sariciftci , Organic Electron. 2012, 13, 919. 10.1016/j.orgel.2012.02.006 PMC3587348 23483783

[145] C. R. Newman , C. D. Frisbie , D. A. da Silva Filho , J. L. Bredas , P. C. Ewbank , K. R. Mann , Chem. Mater. 2004, 16, 4436.

